# A dynamic model of some malaria-transmitting anopheline mosquitoes of the Afrotropical region. I. Model description and sensitivity analysis

**DOI:** 10.1186/1475-2875-12-28

**Published:** 2013-01-23

**Authors:** Torleif Markussen Lunde, Diriba Korecha, Eskindir Loha, Asgeir Sorteberg, Bernt Lindtjørn

**Affiliations:** 1 Centre for International Health, University of Bergen, Bergen, Norway; 2 Bjerknes Centre for Climate Research, University of Bergen/Uni Research, Bergen, Norway; 3 Hawassa University, Hawassa, Ethiopia; 4 National Meteorological Agency of Ethiopia, Addis Ababa, Ethiopia; 5 Geophysical Institute, University of Bergen, Bergen, Norway

**Keywords:** *Anopheles gambiae* complex, Model, Malaria

## Abstract

**Background:**

Most of the current biophysical models designed to address the large-scale distribution of malaria assume that transmission of the disease is independent of the vector involved. Another common assumption in these type of model is that the mortality rate of mosquitoes is constant over their life span and that their dispersion is negligible. Mosquito models are important in the prediction of malaria and hence there is a need for a realistic representation of the vectors involved.

**Results:**

We construct a biophysical model including two competing species, *Anopheles gambiae s.s.* and *Anopheles arabiensis*. Sensitivity analysis highlight the importance of relative humidity and mosquito size, the initial conditions and dispersion, and a rarely used parameter, the probability of finding blood. We also show that the assumption of exponential mortality of adult mosquitoes does not match the observed data, and suggest that an age dimension can overcome this problem.

**Conclusions:**

This study highlights some of the assumptions commonly used when constructing mosquito-malaria models and presents a realistic model of *An. gambiae s.s.* and *An. arabiensis* and their interaction. This new mosquito model, OMaWa, can improve our understanding of the dynamics of these vectors, which in turn can be used to understand the dynamics of malaria.

## Background

This is the first of two papers describing a dynamic model (Open Malaria Warning; OMaWa) of *Anopheles arabiensis* and *Anopheles gambiae s.s.* Our aims in this article are 1) to formulate recent research on the *Anopheles gambiae* complex in a mathematical framework, and 2) to show how the new formulations influence the dynamics of malaria and mosquito populations.

In this paper, we describe a model of the dynamics of the two species and then show how parameters can influence the success of the two species, and how temperature, humidity and mosquito size can influence malaria transmission.

### Climate and malaria

Most of the 149-274 million cases and 537,000-907,000 deaths from malaria occur in sub-Saharan Africa [1,2]. Climate has been one of the main drivers of this disease [3], governing the spatial extent and year-to-year variations. The pathway from climate to malaria goes through the parasite and the mosquito. Although it is well established [4] how parasite development is influenced by temperature [5], the vector’s response to weather and climate is more complex. Mosquito density depends not only on temperature but also on the abundance of breeding sites (rainfall and evaporation) [6], desiccation (humidity) [7], and competition between mosquitoes [8]. In the past 20 years, a shift in the distribution of *An. arabiensis* and *An. gambiae s.s.* has been observed in Kenya [9], showing that the species composition is not static over time. In the context of climate change [10], variability in vector populations is a factor that has not been considered so far.

### Malaria and mosquito models

At the turn of the 20th century the work of several researchers, including Battista Grassi and Ronald Ross, resulted in the discovery that mosquitoes of the *Anopheles* genus transmit malaria [11,12]. Over the next 20 years, Ross, and later Lotka and Waite, developed mathematical models that became central in malaria control [13-19]. In the 1950s, George MacDonald refined these models and showed that DDT could be used to interrupt malaria transmission [20]. Since then, several modelers have followed in the footprints of Ross, Lotka, and MacDonald [21-30]. Some have designed models to show how temperature alone influences malaria transmission [31], while others have focused on the theoretical effect of bed nets [32], multiple interventions [33] or climate change [34-36]. There is also a growing number of models that address the dynamics of immunity within individuals [37] and in communities [21,38].

In 2011, The malERA Consultative Group on Modeling [39] provided a review of the current state of mathematical models and pointed to the importance of good mosquito models for assessing the impact of climate change on malaria.

Many traditional models rely on a threshold principle. The idea has been to find thresholds for longevity, number of bites or days to recovery that must be reduced to interrupt the transmission. With increased computational power it is now possible to make more complex models and hence explore a wider range for the dynamics of malaria and mosquito survival. By integrating the knowledge from simpler models into a complex system, it is possible to test if the assumptions are true over a wider geographical range. In addition, these complex models can make quantitative predictions about strategies for control [40].

### Model summary and motivation

A model is mental copy that describes one possible representation of a system. We present an alternative formulation of the dynamics of *An. gambiae s.s.* and *An. arabiensis*. The model is a system of ordinary differential equations (ODEs) with three compartments: eggs, first to fourth instar larvae, and pupae; an age-structured formulation of adult mosquitoes; and size prediction for adult mosquitoes (measured as wing length in mm). This can be considered the skeleton of the model. As demonstrated later, the model structure can be simplified when mosquito size can be neglected or when we assume no births. The model can be run with a spatial structure in which we include or exclude mosquito dispersion, or as an idealized model in which the model is evaluated at a single point.

The ODEs parametrize daily mortality rates, which are size-dependent for adult mosquitoes; development rates in the aquatic stages; biting rates; fecundity; the probability of finding a blood meal; and mortality related to flushing of eggs, larva and pupa out of oviposition sites. These parametrization schemes are driven by air temperature, relative humidity, relative soil moisture, water temperature, and runoff. As already mentioned, the model can be applied in a spatial domain. In this case, temperature and other environmental data are taken from a regional climate model, the Weather Research and Forecasting Model (WRF) [41]. In the examples shown later, we run the model at a resolution of approximately 50 km and a temporal resolution of 5-20 years in steps of 3 h. In addition to weather data, human [42] and cattle [43,44] densities are introduced to estimate the probability of feeding.

At this spatial resolution, the model should potentially be able to define larger foci of mosquito productivity, while the ability to identify hotspots will be limited [45]. However, 50 km is the standard for regional climate models addressing long-term changes in climate [46]. In addition, the true accuracy of historical cattle and human population density estimates for Africa in general is not likely to be greater than 50 km.

The mosquito model described here is designed to capture the spatial distribution and the time-dependent density of *An. gambiae s.s.* and *An. arabiensis*. If the model can capture the current distribution and density of the two species and how they are related to malaria, a future version of this model, including infections, could be used to explore the long-term impact of current interventions under a changing climate. To have confidence that the model has these abilities, several aspects not considered here should be evaluated (papers under preparation). In addition, if malaria modelers move towards the ensemble thinking widely adopted in the climate community, this model could be one representation of historical and future changes for malaria. The aim of such an ensemble would be to deal with uncertainties in the system. Ultimately, the goal would be to produce policy-relevant information including uncertainty.

We have chosen to represent the non-exponential mortality of *An. gambiae s.s.* and *An. arabiensis* as observed in laboratory settings [47], semi-field conditions [48], and in the field [49]. A common assumption is that in the field, mortality rates are constant with age because of predation [31]. To date, few studies have confirmed this, while there is field-based evidence of age-dependent *Ae. aegypti* mortality [49], which has implications for malaria transmission [50]. In the model, we also describe how mosquito size changes over the season. This might seem to be an overcomplication of the model. The motivation, however, is that we have observed substantial improvements for arid regions such the Sahel when we included mosquito size prediction. Fouet et al. reported that mosquito size is an important adaptation strategy in arid environments [51].

We do not claim that the additional complexity adds any value. Stating this before the model has been fully evaluated and compared to simpler models would be dangerous. The model is thus one possible way of describing the dynamics of *An. gambiae s.s.* and *An. arabiensis*. It is under continuous development, and we expect to add and alter components as new data become available.

To highlight some of the components that contribute to the dynamics of *An. gambiae s.s.* and *An. arabiensis* in the model, five sensitivity experiments focus on the effect of temperature, relative humidity and mosquito size on malaria transmission. We also show how *An. gambiae s.s.* and *An. arabiensis* respond to changes in the probability of finding blood, carrying capacity, initial conditions, and dispersion.

## Material and methods: model description

### Summary of the model

**Figure 1:**
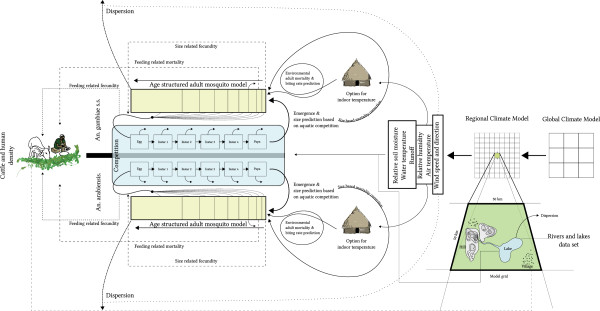
**Overview of the mosquito model.** A (regional) climate model is used to force the mosquito model. In addition, static and semi-static fields are used as part of the parametrization schemes. Human and bovine densities limit the availability of blood meals.

Figure 1 provides an overview of the model. In the following sections we present the ideas behind the model and its general structure, how a climate model is used to drive the mosquito model, and the parametrization schemes used in the model. It should be possible to read each part independently; for example, data from a climate model can be used to drive any malaria model; the parametrization scheme can be used in any malaria model; and the malaria model described here can be used with different parametrization schemes, with or without data from a climate model.

As mentioned above, the model comprises a system of ODEs for eggs, first to fourth instar larvae, and pupae; an age-structured formulation for adult mosquitoes; and size prediction for adult mosquitoes (measured as wing length in mm). The first limitation in the aquatic stage is the availability of ovipositing sites, which is parametrized in terms of relative soil moisture and the potential for puddle formation in a specific location. Once ovipositing sites have been formed, adult female mosquitoes are allowed to deposit eggs until the site is full, defined as the biomass relative to the carrying capacity for the location. To account for density-dependent mortality, first instar larvae can be preyed on by fourth instar larvae [52], and an extra density-dependent mortality term is added to account for prey-independent mortality [53]. The numbers of eggs, larvae and pupae are reduced when the precipitation rate exceeds the infiltration rate. The larval density in the aquatic habitat influences the size of adult mosquitoes [53]. We account for this by predicting mosquito size at emergence as a function of larval density. In addition to temperature and relative humidity [47], mosquito size influences the daily adult survival probability [7,51,54,55] ([56], *Aedes aegypti*). We therefore describe an adult survival model that takes temperature, relative humidity and mosquito size into consideration. In addition, adult mortality and fecundity can increase if there are no or few sources of blood. This follows the idea that a mosquito living in an environment where much energy has to be used to find blood will do this at the cost of survival.

We adopt these general ideas for two species, *An. gambiae s.s.* and *An. arabiensis*. It should be noted that we have less confidence in the model for the *An. gambiae s.s.* M form, since aestivation (as documented by Lehmann et al. [57] and Adamou et al. [58]) is not included. In addition, there are some indications that the M form breeds in larger pools [59] and hence the puddle parametrization might have limited validity for this form.

In addition to time, the model can include two (three, since space is two-dimensional) additional dimensions, namely age and space. The space dimension allows dispersion of mosquitoes, meaning that (re)establishment through migration to areas that were previously free of *An. gambiae s.l.* is possible. The gradual invasion of Brazil by *An. arabiensis* in the 1930s [60] is one example of dispersion.

The ODEs were solved using the ODE solver lsoda [61-63]. The relative and absolute error tolerances were not modified from the original lsoda implementation (1*e*^-6^). The model can be run either as a spatial model (with or without mosquito dispersion) or evaluated at a single point at which movement is neglected. A detailed overview of the possible model parameters can be found in Table 1.

**Table 1:** Model parameters

**Variable**	**Description**	**Equation(s)/reference**
*T*_*indoor*_	Indoor temperature	36
*T*_*air*_	Near surfacetemperature (2 m)	25, 26, 30, 36
*ε*	Potential number ofnew eggs	13
*m*_*n*_	Number of mosquitoes ineach age group	8
*P*(*B*)	Daily probability of gettinga blood meal	41
*T*_*water*_	Water temperature	14, 16, 18
*T*_*soil*_	0-10 cm soil temperature	[91-94]
*β*_*N*,*L*_(*T*_*water*_)	Natural mortiality rate,eggs, larva, and pupa	14, 1, 2, 3, 4, 5, 6
*τ*_*gamb*_	*An. gambiae s.s.* develop- ment rate, aquatic stages	20
*τ*_*arab*_	*An. arabiensis* develop-ment rate, aquatic stages	22
*τ*_*E*_	*An. gambiae s.l.* development rate, eggs	[97] 1
$\tau_{L_{1-4}}$	*An. gambiae s.l.* development rate, instar 1-4	[97] 2, 3, 4, 5
*τ*_*P*_	*An. gambiae s.l.* development rate, pupa	[97] 6
*f*_*arab*_	Aquatic development rate modification *An. arabiensis*	[8]
*f*_*gamb*_	Aquatic development rate modification *An. gambiae s.s.*	[8]
*L*_*n*_	Number of larvae	21, 19
*f*_*arab*_	Mortality rate modification	[72] 17
*f*_*gamb*_	Mortality rate modification	[72] 15
*S*_*f*_	scaling factor for winddispersion	39
*Fr*_*m*_	Flight range	41
*E*	Number of eggs	1
*G*(*T*)	Biting rate/gonotrophiccycle	26
*t*	time	
*B*_*L*_	Larva biomass	1
*β*_*I*,*x*_	Induced mortalityin aquatic and adult stages	1, 2, 3, 4, 5, 6,7, 8
*SM*_*r*_	Dimensionless time varying water constant, or rate at which ovipositing sites are found	24
*K*	Carrying capacity	24
*L*_1_	Number of 1^*st *^instar larva	2
*L*_2_	Number of 2^*nd *^instar larva	3
*L*_3_	Number of 3^*rd *^instar larva	4
*L*_4_	Number of 4^*th *^instar larva	5
*P*	Number of pupa	6
*C*_*pred*_	Predation constant.Currently set to 0	2
*F*_*gonot*_	part of gonotrophic cycle formulation	26
*D*_*d*_	Degree days	[108], 26
*T*_*c*_	Critical temperature	26
*β*_*h*,*m*_	Adult mortality related to feeding	42
*h*	Number of humans	[42]
*Φ*_*ı*,*ȷ*_	flux	39
*n*	Dimension in age grid	
*m*_*size*_	Size of newly emerged mosquitoes	9
$m_{{\text{size}}_{n}}$	Size of mosquitoes in age group *n*	12
*L*_*size*_	Prediction of larva size	10
*a*_*spp*_	Size constant	[22]
*b*_*spp*_	Size constant	[22]
*R*_*p*_	Potential river length in km	23
*Ξ*	Equally spaced riverdataset resolution indegrees	23
*ER*	Earth radius inkm (6371.22)	23
*φ*	latitude in radians	23
*D*	Diffusion coefficient	39
*LT*	Local time	37
*κ*	Diurnal modification fortransport of mosquitoes	37
*HBI*	Human blood index	41, 42
$g\left(m_{{\text{size}}_{n}}\right)$	Size dependent mortality	28
*β*_*N*,*m*_	Natural mortality of adultmosquitoes	32, 7, 8
*ϖ*_*N*,*m*_(*α*,*ζ*,*a*)	Survival curve for adultmosquitoes	35, 31
*α*	Shape parameter for adult survival	3330
*T*_*mod*_	Sub-function forequation 33	34
*ρ*_*bovine*/*cattle*_	Probability of finding cattle	41
*ρ*_*human*_	Probability of findinghumans	41

Description of model components.

### Differential equations for the aquatic compartment

The aquatic compartment consists of six stages: eggs (*E*), four larval stages (*L*_1_,*L*_2_,*L*_3_,*L*_4_), and pupae (*P*). Transitions between the different compartments can be expressed in terms of delayed equations. To simplify the solution and avoid numerical instabilities, we approximate the model as ODEs [21]. Lunde et al. reported on the errors introduced by this approximation [64].

New eggs added to the population depend on the number of adult mosquitoes (*m*), the size of adult mosquitoes (*m*_*size*_), the inverse length of the gonotrophic cycle (*G*(*T*)), how much water is available (*SM*_*r*_, dimensionless) and the larval biomass already present in puddles (*B*_*L*_):

(1) $$\begin{array}{cc} \frac{\mathrm{\delta E}}{\mathrm{\delta t}} & =\varepsilon \left(m,m_{{\text{size}}_{n}}\right)\cdot G(T)\cdot SM_{r}\cdot \left(1-\frac{B_{L}}{K}\right)\\ & \;-\left(\beta_{N,E}(T)+\beta_{I,E}+\tau_{E}(T)\right)\cdot E, \end{array}$$

where $\varepsilon \left(m,m_{{\text{size}}_{n}}\right)$ represents potential new eggs from each age group, *G*(*T*) is either constant or dependent on temperature *T*, *SM*_*r *_is a function of the relative soil moisture and the potential puddle formation area, *K* is the maximum larval biomass a grid cell can hold, *β*_*N*,*E*_(*T*) is natural mortality rate for eggs [Eqs. (16) and (18)], *β*_*I*,*E *_is the induced mortality rate for eggs (not specified) and *τ*_*E *_is the inverse of development time from eggs to first instar larvae.

The term 1-*B*_*L*_/*K* is used as a scaling factor to modify the growth rate. When the population is low compared to the breeding sites available, its growth is high. As the population grows, there is more competition for food, predators become more abundant, and the growth slows. In the egg compartment this represents the idea that the mosquitoes will lay fewer eggs when breeding sites are already occupied [65].

First instar larvae (*L*_1_) are added as eggs develop into larvae. Additional mortality is added in the transition stage in relation to how much biomass there already is in a given location [53]. This approximation of increased (density-dependent) mortality arises because of competition and predators; if a puddle already is full, the number of eggs developing to first instar larvae is reduced, whereas if a puddle is empty (1-*B*_*L*_/*K *= 1), no extra mortality occurs. Similar terms could have been added to the second, third and fourth instar larvae, but we assume that earlier life stages will be affected more by density-dependent competition and predation.

Shoukry looked at how fourth instar larvae of *An. pharoensis* prey on first instar larvae during a 24-h experiment [52]. Using these data, we add additional mortality for first instar larvae according to the density of fourth over first instar larvae. The constant *C*_*pred *_is tunable to both limit the predation on *L*_1 _and make it more specific to species in the future. At most temperatures, this constant does not influence the density of mosquitoes (Additional file 1).

The number of first instar larva is given by:

(2) $$\begin{array}{ll} \frac{\delta L_{1}}{\mathrm{\delta t}} & =\tau_{E}(T)\cdot E\cdot \left(1-\frac{B_{L}}{K}\right)-\left(\beta_{N,L}(T)+\beta_{I,L}+\tau_{L_{1}}(T)\right)\\ & \;\cdot L_{1}-\frac{0.4465}{{\left(\frac{L_{4}}{L_{1}}+1\right)}^{2.9891}}\cdot C_{\text{pred}}. \end{array}$$

Second (*L*_2_), third (*L*_3_) and fourth instar larvae (*L*_4_) and pupae (*P*) are controlled by the development rate *τ* and mortality *β*:

(3) $$\frac{\delta L_{2}}{\mathrm{\delta t}}=\tau_{L_{1}}(T)\cdot L_{1}-\left(\beta_{N,L}(T)+\beta_{I,L}+\tau_{L_{2}}(T)\right)\cdot L_{2}$$

(4) $$\frac{\delta L_{3}}{\mathrm{\delta t}}=\tau_{L_{2}}(T)\cdot L_{2}-\left(\beta_{N,L}(T)+\beta_{I,L}+\tau_{L_{3}}(T)\right)\cdot L_{3}$$

(5) $$\frac{\delta L_{4}}{\mathrm{\delta t}}=\tau_{L_{3}}(T)\cdot L_{3}-\left(\beta_{N,L}(T)+\beta_{I,L}+\tau_{L_{4}}(T)\right)\cdot L_{4}$$

(6) $$\frac{\mathrm{\delta P}}{\mathrm{\delta t}}=\tau_{L_{4}}(T)\cdot L_{4}-\left(\beta_{N,P}(T)+\beta_{I,P}+\tau_{P}(T)\right)\cdot P,$$

where *β* is the daily mortality rate, with the first subscript denoting natural (N) or induced (I) mortality and the second subscript denoting the aquatic stage. The subscript for the development rate, *τ*, corresponds to the aquatic stage. The parametrization schemes and data sources used to estimate the rate at which eggs are laid (*G*(*T*) and *ε*), mortality (*β*) and the development rate (*τ*) are discussed later.

### Differential equations for adult mosquitoes

The life history and mortality rate vary over the lifespan of a mosquito population. We formulated a model to account for this variation. Adult mosquitoes are denoted by *m*_*n*_, where *n* indicates the age group; *n *= 1 is the youngest group and *n *= 9 refers to the oldest mosquitoes. The age groups in the model are *m*_1 _=[0,1], *m*_2 _= (2,4], *m*_3 _= (5,8], *m*_4 _= (9,13], *m*_5 _= (14,19], *m*_6 _= (20,26], *m*_7 _= (27,34], *m*_8 _= (35,43] and *m*_9 _= (44,*∞*] days, with ageing coefficients *a*_*n *_of 1.000, 0.500, 0.333, 0.250, 0.200, 0.167, 0.143, 0.125 and 0.067 for n=1,2,…,9, respectively. Mosquito ageing is represented by *Ψ*_*n*_, where *n* denotes the age group. Ageing is time-invariant and is thus not related to the number of gonotrophic cycles.

Although there is no ageing from age group 9, the term *Ψ*_9 _is included to limit the concentration of old mosquitoes. This is a user-specified variable and in the model results shown here we set this to $\frac{1}{15}\text{da}y^{-1}$ for *An. arabiensis* and *An. gambiae s.s.*; this value should be set to ensure that mosquito populations can survive during dry periods [66,67], but still hinder accumulation of old mosquitoes. This can be particularly useful if the mortality model described later is replaced with a model in which mortality is independent of age.

When *m* is written with subscripts *ı* and *ȷ* in addition to *n*, this denotes inclusion of mosquitoes from neighboring areas. For example, subscript *ı*-1 indicates that mosquitoes to the west of the point of interest are interacting with the point of interest. The formulation presented here includes movement of mosquitoes, and where appropriate we denote mosquitoes by *m*_*n*,*ı*,*ȷ*_.

Again, *β* denotes mortality, with the first subscript denoting natural (N) or induced (I) mortality and the second subscript denoting the age group (*m*_*n*_) of the mosquitoes. *Φ* represents the mosquito flux (transport) and subscripts *ı* and *ȷ* define which boundaries are evaluated. This is discussed in the section “Movement of mosquitoes”.

The number of adult mosquitoes of a specific age in a grid point is controlled by new mosquitoes from *m*_*n*-1_, as well as the flux to and from the point of interest $\left(\overset{1}{\underset{ı=-1}{\sum }}\overset{1}{\underset{ȷ=-1}{\sum }}\Phi_{ı,ȷ}m_{n,ı,ȷ}\right)$, natural mortality $\beta_{N,m_{n}}$, induced mortality $\beta_{I,m_{n}}$, ageing to *m*_*n*+1_, and mortality due to lack of food (*P*(*B*)). Parametrization schemes related to mortality are discussed later.

This results in the following equation for the first age group:

(7) $$\begin{array}{ll} \frac{\delta m_{1}}{\mathrm{\delta t}} & =\tau_{P}(T)\cdot P+\overset{1}{\underset{ı=-1}{\sum }}\overset{1}{\underset{ȷ=-1}{\sum }}\Phi_{ı,ȷ}m_{1,ı,ȷ}\\ & \;-\left(\beta_{N,m_{1}}+\beta_{I,m_{1}}+\Psi_{1}\right)\cdot m_{1}. \end{array}$$

The equations for age groups *n *= [2,9] are

(8) $$\begin{array}{ll} \frac{\delta m_{n}}{\mathrm{\delta t}} & =\Psi_{n-1}\cdot m_{n-1}+\overset{1}{\underset{ı=-1}{\sum }}\overset{1}{\underset{ȷ=-1}{\sum }}\Phi_{ı,ȷ}m_{n,ı,ȷ}\\ & \;-\left(\beta_{N,m_{n}}+\beta_{I,m_{n}}+\Psi_{n}+\beta_{h,m}\right)\cdot m_{n}. \end{array}$$

### Differential equations predicting mosquito size

Mosquito size (*m*_*size*_) is important for the efficiency of mosquito multiplication. There are also some indications that increased body size is a strategy for survival in arid environments [7]. In general, high larval density leads to a smaller body size as adults, and vice verse [68]. Where only one species is competing for a resource, such as in a small puddle, mosquito size, and hence the number of eggs laid by each mosquito, will be of less importance. If two species are competing for the same resource (e.g. *An. arabiensis* and *An. gambiae s.s.*), the trade off between development time and size can be important in competition for breeding sites. *An. gambiae s.s.* generally develop faster than *An. arabiensis*, but end up with a smaller body size. *An. arabiensis* spends more time in the aquatic stages and develops larger bodies, and can thus produce more eggs. Since our model includes competition between those species, we describe mosquito size as a function of competition for breeding sites. In theory this should improve our ability to separate geographical and seasonal distributions of *An. arabiensis* and *An. gambiae s.s.*

Since the size of *An. arabiensis* and *An. gambiae s.s.* stabilizes after approximately 4 days [7] and ovoposition does not start before this, it is not necessary to differentiate the maximum and minimum size depending on age to mimic changes in the number of eggs per mosquito with age. However, this may be required if mortality based on desiccation [7,69] is used. Although mosquito size at a given time can be approximated using finite differences, we develop a different approach that is more efficient in terms of computational time in our model framework. Mosquito size for the first age group depends on larval size. Since the pupation time is short, this assumption is justified, although it might introduce minor errors. In a future version of the model, we plan to predict larval size dynamically. The limitations set on mosquito size (described in “Parametrization schemes in the aquatic stages”) in this model might lead to *An. arabiensis* that are slightly too small compared size in the field study of Ye-Ebiyo et al. [70], but the size is in line with studies by Huestis et al. [71] and Kirby et al. [72]. Kirby et al. also noted that mixed populations of *An. arabiensis* and *An. gambiae s.s.* had a negative effect on mosquito size at some temperatures. This mechanism is not included in the current work. However, the most important aspect of modelling of mosquito size is to capture seasonal and spatial variations.

For size prediction we use the symbol $m_{{\text{size}}_{n}}$, where *n* is the age group as described above.

The size (wing length in mm)of newly emerged mosquitoes is approximated according to the linear relationship

(9) $$m_{{\text{size}}_{e}}=1.25+5\cdot L_{\text{size}},$$

where larva size *L*_*size *_(in mg) is approximated as:

(10) $$L_{\text{size}}=a_{\text{spp}}-b_{\text{spp}}\cdot \text{min}\left(\frac{B_{L}}{K},1\right).$$

The constants *a*_*spp *_and *b*_*spp *_are 0.45 and 0.12 for *An. arabiensis* and 0.383 and 0.147 for *An. gambiae s.s.*, respectively [22].

The size of mosquitoes in the first age group at any time is given by

(11) $$\begin{array}{ll} \frac{\delta m_{{\text{size}}_{1}}}{\mathrm{\delta t}} & =\text{min}\left(\text{max}\left(\frac{\tau_{P}(T)\cdot P}{m_{1}},0\right),1\right)\\ & \;\cdot \text{log}\left(\frac{m_{{\text{size}}_{e}}}{m_{{\text{size}}_{1}}}\right)\cdot m_{{\text{size}}_{1}}. \end{array}$$

Therefore, the size of newly emerged mosquitoes ($m_{\text{siz}e_{1}}$) depends on the number of newly emerged pupae and the relative density of larva at the breeding site.

For the remaining age groups, size $m_{{\text{size}}_{n}}$ is estimated as

(12) $$\frac{\delta m_{{\text{size}}_{n}}}{\mathrm{\delta t}}=\text{min}\left(\frac{\Psi_{n-1}\cdot m_{n-1}}{m_{n}},1\right)\cdot \text{log}\left(\frac{m_{{\text{size}}_{n-1}}}{m_{{\text{size}}_{n}}}\right)\cdot m_{{\text{size}}_{n}}.$$

Therefore, the size in age groups 2-9 only depends on the number of mosquitoes surviving from one age group to the next (*m*_*n*-1_) and the size of mosquitoes in the younger age group ($m_{{\text{size}}_{n-1}}$).

### Model forcing

To drive a dynamic malaria model it is necessary to have boundary conditions that are consistent over time and space. Temperature, relative humidity, and rainfall data from weather stations are point measures. Hence, they might not be representative of larger areas over shorter time scales. This is especially true in areas with varying topography or where convective rainfall is dominant [73-75]. Despite the limitations of rainfall stations, they can provide a robust estimate of large-scale events. By pooling data from several stations, the error for a single station is reduced and the data can provide a good estimate for dry and wet years, for example. Hence, weather stations are useful tools for validating climate models.

The problems of point measurements are described later, and represent one of the reasons why OMaWa is tightly linked to a climate model. As shown in sensitivity experiments, the model can also be run with constant forcing (e.g. temperature) or with data from weather stations.

Where we present results for Africa as a whole, OMaWa is driven by data from WRF 3.3.1. This realization (TC50), described in part two of this paper, has a tropical channel set-up in which set-up, the domain consists of boundaries above and below a certain latitude and no side boundaries. The model was run at 50-km resolution from January 1, 1989 to January 1, 2009. At the northern (45°N) and southern (-45°N) boundaries the model was driven by Era Interim. The Kain Frisch cumulus parametrization scheme was used [76,77]. This experiment was not designed to reproduce observed year-to-year weather variability, but to assess the mean mosquito density and distribution. The driving experiment is described in the section on model validation.

### Climate and weather models

Currently, our best guess of (future) climate at multidecadal time scales comes from general circulation models (GCMs). These models are designed to close the energy budget of the Earth and include an interactive representation of the atmosphere, ocean, land, and sea ice. A set of scenarios with different emissions describes how sensitive the climate is to atmospheric constituents (greenhouse gasses) [78]. While climate is the average weather over time and space, weather can change over minutes, hours, days and seasons. The same equations used to predict climate are used to predict weather. However, weather forecasts are more dependent on current observations of the atmosphere. Hence, weather predictions are initial value problems, whereas climate simulations are rather boundary value problems.

Both climate and weather models are mostly structured on a grid, with coordinates from west to east (*x*), north to south (*y*) and bottom to top (*z*). In the grid, one square (or polygon) represents the weather within that square. While climate models often have a horizontal resolution of more than 10000 *km*^2^, operational weather models such as the European Centre for Medium-Range Weather Forecast (ECMWF) model are run at approximately 160 *km*^2^. If the state of the atmosphere is observed correctly, higher resolution can lead to better local skill in predicting the weather. A hybrid between a weather model and a climate model is a limited-area model (LAM), which relies on initial and boundary conditions from a weather or climate model. Given these conditions (weather), the LAM can be run at a higher resolution over a limited area, which potentially improves the spatial accuracy of the coarse model [79]. The WRF model is a widely used LAM [41].

In tropical regions, most rainfall comes from convective clouds. This type of rainfall is generally intense and of short duration. The geographical extent of such rainfall episodes may be limited. Therefore, rainfall measurements in regions where convective rainfall is dominant should be handled with care [74,75,80,81], especially when extrapolating station data to areas with no data. While station data are accurate at a specific point, climate models and satellite estimates give a more general description of the weather within a certain area; Chen and Knutson reviewed how models compare to observations at varying scales [82]. Since future climate is projected using climate models and considering the limitations of weather stations, construction of a mosquito/malaria model around a LAM is a good choice. The LAM will have higher resolution than most climate models, with higher-resolution orography, coastlines, and land use, but will still give a general description of the weather within a certain area.

### Parametrization schemes in the aquatic stages

To relate a variable such as mortality to the physical environment, we need simplified equations that describe this relationship. An equation in which temperature influences mortality only states that there is a relationship between the two, but does not explain why temperature modifies mortality. In this paper we use parametrization schemes to represent the influence of the environment on mosquitoes. This section describes the aquatic parametrization schemes used, excluding water availability, which is discussed later.

The aquatic stages comprise eggs, four instar stages, and pupae. The number of eggs in a location at any time is controlled by the number of potential new eggs laid (*ε*), available water (*K*), natural and induced mortality (*β*_*N*/*I*,*E*/*L*/*P*_) and movement from the *E* to the *L*_1 _compartment. In addition, 20% instant mortality is introduced when rainfall exceeds the infiltration rate. This is in line with observations by Paaijmans et al. [83]. The number of new eggs is simplified to a function of the number of gravid mosquitoes in each age group and their size (measured as wing length) based on observations [55,84-86]. The critical size is set to a wing length of 2.6 mm, which is less than that observed by Lyimo and Takken [85] but greater than observations by Yaro et al. [87]. Maximum wing length is set to 3.3 mm for *An. gambiae s.s.*[88,89] and 3.7 mm for *An. arabiensis*[70]. The relationship between the number of eggs (*ε*) and wing length $\left(m_{{\text{size}}_{n}}\right)$ is then approximated according to the linear relationship

(13) $$\begin{array}{c} \varepsilon \;=\;\overset{9}{\underset{n=1}{\sum }}\left\{\begin{array}{cc} \left(-433.3+166.7\cdot m_{{\text{size}}_{n}}\right)\cdot m_{n} & \;\text{if}\;m_{{\text{size}}_{n}}>2.6\;\text{mm}\\ 0 & \;\text{otherwise} \end{array}\;\right\}\;, \end{array}$$

where *m*_*n*_ is the number of mosquitoes in age group *n*. Note that this limits the number of eggs laid by a single mosquito per gonotrophic cycle to approximately 184, which is somewhat less than the number observed by Yaro et al. [87], but in line with that reported by Howard et al. [90].

#### Estimation of water temperature

Using the 0-10-cm soil temperature (*T*_*soil *_) from the NOAH land surface model [91-94] to approximate the mean water temperature (*T*_*water *_) in larval habitats, we assume that evaporative cooling and heat fluxes at the water boundaries are negligible. Hence, the water temperature is equal to the top soil temperature. Paaijmans et al. showed that the 5-cm soil temperature represents the water temperature in small ponds reasonably well [95]. Therefore, the model will have limited validity in areas where larger puddles are the main breeding sites. There is also a chance that diurnal fluctuations will be slightly over- or underestimated. When a grid cell covers several *km*^2^, this effect should be negligible, although we do not have data to support this. We hope to improve the prediction of water temperature in the future, either by modelling this explicitly or using a parametrized version based on data from Huang et al. [96].

#### Parametrization of mortality

We used two approaches to calculate mortality in the aquatic stages. In the simpler approach, we assume that mortality and development time in the aquatic stages are independent of the species. We also assume that the relationship between the mortality rate and temperature is the same for eggs, instars and pupae. In this method we do not consider competition effects as described by Paaijmans et al. [8]. This type of parametrization is suitable when the model is used for one species only (e.g. if the model represents an area where only one of the two species is present).

#### Species-independent mortality (BLL)

**Figure 2:**
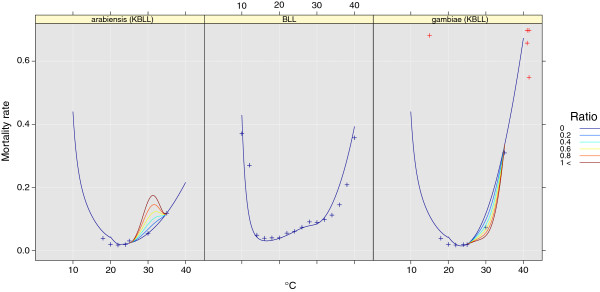
**Water temperature and mortality rates (*day***^***-1***^**) in the aquatic compartments.** Blue points show data used to estimate the mortality curves. Blue lines indicate mortality without competition, while light blue to red shows mortality as competition increases. For reference, red points show data from Holstein [98].

Data provided by Bayoh and Lindsay [97] were used to describe the mortality rate according to Eq. (14) (*p *< 0.01, *R*^2 ^= 0.81). We call this the BLL method. Mortality rate data are plotted in Figure 2b.

(14) $$\begin{array}{ll} \beta_{N,L}\left(T_{\text{water}}\right) & =\left(\frac{k_{1}}{T_{\text{water}}^{k_{2}}}+e^{k_{3}\cdot T_{\text{water}}-k_{4}}\right)\\ & \;\cdot k_{5}+\frac{k_{6}}{1+k_{7}\cdot e^{k_{8}\cdot \left(T_{\text{water}}-k_{9}\right)}}, \end{array}$$

where *β*_*N*,*L*_(*T*_*water*_) = *β*_*N*,*E*_(*T*_*water*_) = *β*_*N*,*P*_(*T*_*water*_) is the aquatic mortality rate per day and *T*_*water *_is the water temperature (°C). The constants *k*_*n *_are given in Table 2.

**Table 2:** Constants for equation 14 and 33

**Constant**	**Value**	**Equation**
*k*_1_	700000	14
*k*_2_	8.4	14
*k*_3_	.126	14
*k*_4_	10.8	14
*k*_5_	150	14
*k*_6_	-.08	14
*k*_7_	.1	14
*k*_8_	-.61	14
*k*_9_	33	14
*c*_1_	0.1675256	33
*c*_2_	0.0121402	33
*c*_3_	0.1686	33
*c*_4_	1.991	33
*c*_5_	1.881	33
*c*_6_	4.641589*e*26	33
*c*_7_	250	33
*c*_8_	23	33
*c*_9_	12	33
*c*_10_	100	33
*c*_11_	3	33

#### Species-dependent mortality (KBLL)

Kirby et al. reported that the mortality rate of *An. gambiae s.s.* and *An. arabiensis* is modulated by the presence of each other in the temperature range 25-35°C [72]. To account for this we developed two mortality models, one for *An. gambiae s.s.* and one for *An. arabiensis*. We call this parametrization scheme KBLL. The mortality rates are based on data from Bayoh and Lindsay [97] and from Kirby et al. [72]. Although Holstein also reported larval mortality for (*An. gambiae s.s.*) when exposed to extreme low and high temperatures [98], we did not include these data when estimating the mortality curves. However, the data are plotted in Figure 2 for comparison. According to our curves, the *An. arabiensis* mortality rate will increase in the range 25-35°C as the relative presence of *An. gambiae s.s.* increases. Conversely, the mortality rate of *An. gambiae s.s.* will decrease as the proportion of *An. arabiensis* increases. The mortality rate *β*_*N*,*L *_is given by

(15) $$F_{\text{arab}}=\text{min}\left(\frac{\overset{4}{\underset{n=1}{\sum }}L_{n,\text{arab}}}{\overset{4}{\underset{n=1}{\sum }}L_{n,\text{gamb}}},1\right)$$

(16) $$\begin{array}{l} \beta_{N,L,\text{gamb}}\left(T_{\text{water}}\right)=\left\{\begin{array}{llll} & & \;0.002404075\cdot T_{\text{water}}^{2}-0.1127944\cdot T_{\text{water}}+1.337783\\ \beta_{N,L}\left(T_{\text{water}}\right) & \cdot & {\left(0.4+0.6\cdot \left(1+\sin\left(-10.9956+0.3142\cdot T_{\text{water}}\right)\right)\right)}^{F_{\text{arab}}} & \;\text{if}\;25\leq T_{\text{water}}\leq 35 \end{array}\right) \end{array}$$

and

(17) $$F_{\text{gamb}}=\text{min}\left(\frac{\overset{4}{\underset{n=1}{\sum }}L_{n,\text{gamb}}}{\overset{4}{\underset{n=1}{\sum }}L_{n,\text{arab}}},1\right)$$

(18) $$\begin{array}{l} \beta_{N,L,\text{arab}}\left(T_{\text{water}}\right)=\left\{\begin{array}{cc} 0.0006556736\cdot T_{\text{water}}^{2}-0.02980226\cdot T_{\text{water}}+0.3587285\\ \beta_{N,L}\left(T_{\text{water}}\right)\cdot {\left({\left(2+\cos\left(-18.8496+0.6283\cdot T_{\text{water}}\right)\right)}^{0.9508002}\right)}^{F_{\text{gamb}}} & \;\text{if}\;25\leq T_{\text{water}}\leq 35\\ \beta_{N,L,\text{gamb}}\left(T_{\text{water}}\right) & \;\text{if}\;T_{\text{water}}\leq 21.91209. \end{array}\right) \end{array}$$

*f*_*gamb *_and *f*_*arab *_are the ratio of *An. gambiae s.s.* to *An. arabiensis* larvae and *An. arabiensis* to *An. gambiae s.s.* larvae, respectively. At each time step, *L*_*size *_is estimated as a function of *B*_*L *_and *K*. As the density increases, there will be more competition and hence less food for each larva, which leads to smaller larvae.

#### Parametrization of the development rate

**Figure 3:**
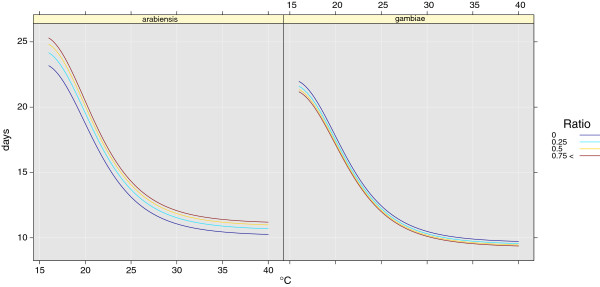
**Water temperature according to development time in days from first instar to adult.** Left panel: ratio of *An. gambiae s.s.* to *An. arabiensis*. When greater numbers of *An. gambiae s.s.* are present, *An. arabiensis* develop more slowly. Right panel: ratio of *An. arabiensis* to *An. gambiae s.s.*. When greater numbers of *An. arabiensis* are present, *An. gambiae s.s.* develop more quickly.

The rate of development between the different aquatic stages follows the corrected version of Bayoh and Lindsay [97]. Since these data are only valid for *An. gambiae s.s.*, we made a small modification to prolong the development times for *An. arabiensis*. Data from Kirby et al. [72] and Paaijmans et al. [8] suggest that time for development from a larva to an adult is approximately 5.5% longer for *An. arabiensis* than for *An. gambiae s.s.* Hence, we increased the development time for *An. arabiensis* by 5.5%. The reason for this longer development time is that *An. arabiensis* takes longer to develop a larger body. Curves of the development rate are shown in Figure 3.

The two previous studies also suggest that the development rate [8] and mortality [72] of the two species are modulated by the presence of each other, so we take account of this in out model. The development time for *An. arabiensis* is prolonged in the presence of *An. gambiae s.s.*, while the time is shortened for *An. gambiae s.s.* as the relative proportion of *An. arabiensis* increases. Using data from Paaijmans et al. [8], the development rate *τ* is modified according to

(19) $$f_{\text{arab}}=\text{min}\;\left(100\cdot \frac{\overset{4}{\underset{n=1}{\sum }}L_{n,\text{arab}}}{\overset{4}{\underset{n=1}{\sum }}L_{n,\text{gamb}}+\overset{4}{\underset{n=1}{\sum }}L_{n,\text{arab}}},75\right)$$

(20) $$\tau_{\text{gamb}}=\tau_{\text{gamb}}\cdot {\left(1-f_{\text{arab}}\cdot 0.0008421\right)}^{-1}$$

for *An. gambiae s.s.* and

(21) $$f_{\text{gamb}}=\text{min}\;\left(100\cdot \frac{\overset{4}{\underset{n=1}{\sum }}L_{n,\text{gamb}}}{\overset{4}{\underset{n=1}{\sum }}L_{n,\text{gamb}}+\overset{4}{\underset{n=1}{\sum }}L_{n,\text{arab}}},75\right)$$

(22) $$\tau_{\text{arab}}=\tau_{\text{arab}}\cdot {\left(1+f_{\text{gamb}}\cdot 0.002138\right)}^{-1}$$

for *An. arabiensis*. *f*_*arab *_ and *f*_*gamb *_is the fraction of *An. arabiensis* and *An. gambiae s.s.*, respectively.

### Parametrization of breeding sites

The formation of puddles can be described as a balance of runoff, infiltration, evaporation, and rainfall entering the puddle. The formulation of an idealized puddle can be found in Additional file 2.

Modelling of every single breeding site requires high enough resolution to resolve the puddle. In practice this is not possible and the problem has to be simplified.

Mushinzimana et al. described typical breeding sites in a Kenyan highland area [99]. Most of the puddles were located at less than 100 m from rivers, which means we can assume that semi-permanent puddles will mostly form in the proximity of rivers and lakes. They also found that the number of breeding sites was close to threefold higher in the rainy season compared to the dry season, and grouped breeding sites by surface area.

If we assume that breeding mainly occurs in the vicinity of potential rivers and lakes, the availability of breeding sites can be expressed as a function of potential river length and soil saturation. At high resolution this might not always be true [6], but since the model is designed to be applied to coarser grids, we believe the assumption is as reasonable as or more reasonable than the common assumption that puddle formation is only dependent on rainfall [29]. The newest version of the NOAH land surface model in WRF 3.4 also includes groundwater and dynamic vegetation, and future versions might change the way in which puddles are parametrized. In OMaWa we introduce a simple parametrization scheme to represent breeding sites.

The Hydrological Data and Maps based on SHuttle Elevation Derivatives at Multiple Scales (HydroSHEDS) 15s river data set from the US Geological Survey (USGS) [100] was used to derive the total potential river length within a grid cell. Since the algorithm used to develop this data set describes where water would collect if it were available within the catchment, it also represents a general description of the potential for water aggregation within an area. However, the validity might decrease on moving to finer scales [6].

Here we divide rivers into three different classes: perennial, intermittent and ephemeral streams. For each class, potential river length (*R*_*p*_, km) within a grid is defined as

(23) $$R_{p}=\sum \Xi \cdot \frac{2\mathrm{\Pi ER}}{360}\cdot \cos\varphi ,$$

where Ξ is the equally spaced river data-set resolution in degrees, where *Δlon *= *Δlat*, *ER* is the radius of the Earth (6371.22 km) and *φ* is latitude in radians.

In a simplified model we estimate puddle volume as a function of river length and relative soil moisture. Although this is a very crude estimate, we compared this simple model with data from Mushinzimana et al. [99] and derived a simple expression for the carrying capacity in a grid cell:

(24) $$K=\frac{B_{L,\text{max}}}{km_{\text{river}}}\cdot R_{p}\cdot SM_{r},$$

where $\frac{B_{L,\text{max}}}{km_{\text{river}}}$ is the maximum larval biomass per km of river (2400 mg, estimated from data collected by Munga et al. [101]) and *SM*_*r *_is the relative soil moisture content (fraction).

In the current implementation we do not distinguish between fast- and slow-flowing rivers. It should be noted that this way of approximating breeding sites has limited validity in areas with irrigation or around rivers where breeding sites could form as rivers recede [66,67,102]. Some special cases, such as along the River Nile in Sudan, where breeding sites form as a result of rainfall hundreds of kilometers away, will not be captured at all [103].

### Parametrization of the gonotrophic cycle

**Figure 4:**
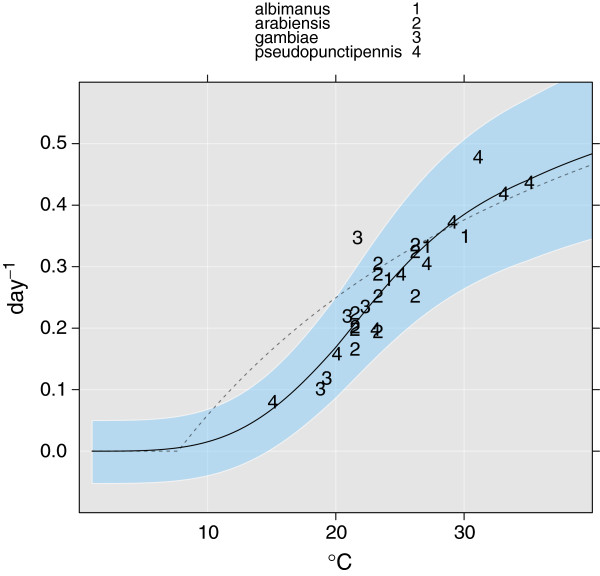
**Inverse of the duration of the gonotropic cycle according to the mean daily temperature (in °C).** The solid black line shows Eq. 26 and the dashed line shows the formula given by Hoshen and Morse [108].

The gonotrophic cycle depends on temperature and is important for the vectorial capacity of mosquitoes. Lardeux et al. studied the gonotrophic cycle for *An. pseudopunctipennis*[104]. We combine their data with other published studies on anophelines to estimate the length of the gonotropic cycle. There are few studies on *An. gambiae s.l.*, and hence we have to assume that other anophelines share the same physiology and strategy with respect to the gonotropic cycle. Ruiz et al. showed there are some differences [23], but until further evidence of the reproductive strategies of different members of *Anopheles* genera becomes available, we will not consider this effect. Studies used to develop the formula include those by Guillermo et al. ([105], *An. albimanus*), Afrane et al. ([106], *An. gambiae s.l.*), and Maharaj ([107], *An. arabiensis*). We also include the formula given by Hoshen and Morse [108]. Their model is based on degree days and is included according to Eq. (26). The gonotropic rate (*day*^-1^) and data used to develop the formula are shown in Figure 4.

(25) $$\begin{array}{c} F_{\text{gonot}}=\text{min}\;\left(\text{max}\;\left(-\frac{2}{3}+\frac{1}{30}\cdot T_{\text{air}},0\right),.5\right) \end{array}$$

(26) $$\begin{array}{ll} G(T) & ={\left(1+\frac{D_{d}}{T_{\text{air}}-T_{c}}\right)}^{-1}\\ & \;\cdot F_{\text{gonot}}+{\left(1.71+544347.6\cdot T_{\text{air}}^{-3.93}\right)}^{-1}\cdot \left(1-F_{\text{gonot}}\right), \end{array}$$

where *T*_*air *_is the air temperature (°C), *D*_*d *_is degree days, and *T*_*c *_is the critical temperature from Hoshen and Morse [108], with *D*_*d *_= 37, and *T*_*c *_= 7.7.

### Parametrization of the age-dependent mortality of adult mosquitoes

**Figure 5:**
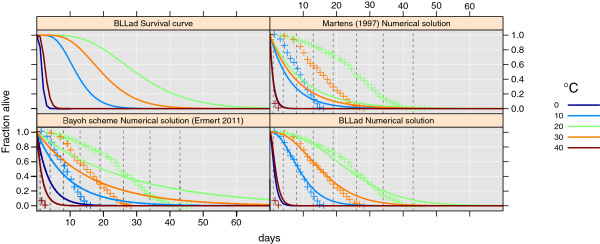
**Proportion of *An. gambiae s.s. *surviving at 60% relative humidity and mean temperature of 0, 10, 20, 30, and 40°C (selected for clarity) according to time (in days).** Dashed vertical lines indicate the different age groups in the model. The Survival curve panel shows Eq. 31, while the Numerical solution panel shows survival in the model when the age groups are split into nine classes. For reference we also show survival according to the Marten equation (27) and Eq. 7 from Ermert et al. (Bayoh scheme) [29]. The mean absolute error for all combinations of temperature and relative humidity was 73 for our model, 171 for the Marten model, and 129 for the Bayoh scheme.

The mortality of adult anophelines differs according to age and species [7,107,109]. This has often been overlooked in mosquito models [23,110]. To show how this assumption can influence the stability of mosquito populations and malaria transmission, we use the mortality model of Martens [110] as a reference. We also plot Eq. 7 from Ermert [29] in Figure 5 to highlight the differences between this model and established models. For convenience, we repeated Marten’s equation, as follows:

(27) $$\beta_{N,m}(T)=1-e^{-\frac{1}{-4.4+1.31\cdot T-.03\cdot T^{2}}}.$$

Our new survival curves are based on unpublished data from Bayoh and Lindsay [47]. The validity ranges from 5 to 40°C by 5°C and 40-100*%* by 20% relative humidity. We name the scheme BLLad (Bayoh-Lindsay-Lunde adult mortality). The data set and the curves are valid for *An. gambiae s.s.* The lowest agreement between the model and the data is at 40% relative humidity and 40°C. While the data suggest that all *An. gambiae s.s.* would be dead after approximately 2 days, the survival curve would result in no mosquitoes after approximately 4 days at 40% relative humidity and 40°C. To correct for this error, we include data from Kirby and Lindsay [111], who described the responses of *An. gambiae s.s.* and *An. arabiensis* to high temperatures. By assuming that maximum survival is 480 min for *An. gambiae s.s.* and 1440 min for *An. arabiensis* at temperatures greater than 40°C, we can set the mortality rate to 3*day*^-1^ and 1*day*^-1^, independent of age group. However, there are uncertainties at relative humidity below 40%. The lack of studies in this range is a limitation of this survival model, and could make the model less accurate for *An. gambiae s.l.* in some regions. The basic principle of these survival curves is that mortality will be low in the first few days after emergence. In addition, mosquitoes that survive up to a certain age have a higher survival probability (depending on *T*_*air *_and relative humidity). In Figure 5, survival at 60% relative humidity and 0, 10, 20, 30, and 40°C is plotted.

Size affects the survival of adult mosquitoes [7,51,54,55] ([56], *Aedes aegypti*). If we assume that the major differences in mortality between *An. gambiae s.s.* and *An. arabiensis* can be attributed to mosquito size, we can modify *α* as a linear function of mosquito size. Here we subjectively choose reasonable constants for *h*(*m*_*size*_). *T*_*air *_may be completely or partly replaced by indoor temperature (*T*_*indoor *_, described later), depending on the proportion of mosquitoes indoors. In experiments covering the African domain, we assumed that 80% of *An. gambiae s.s.* and 20% of *An. arabiensis* are located indoors.

(28) $$g\left(m_{\text{siz}e_{n}}\right)=2.1731-0.3846\cdot m_{\text{size}}$$

(29) $$f(\text{RH})=6.48007+0.69570\cdot (1-e^{-0.06\cdot \text{RH}})$$

(30) $$\;\begin{array}{ll} \alpha & =g\left(m_{\text{size}}\right)\\ & \;\times e^{10+{\left(1+\frac{T_{\text{air}}+1}{21}\right)}^{\left(2/3\right)}\cdot \left({\left(1+\frac{\left(T_{\text{air}}+1\right)}{21}\right)}^{2}-\left(1+\frac{T_{\text{air}}+1}{21}\right)\cdot 2-f\;(\text{RH})\right)} \end{array}$$

(31) $$\varpi_{N,m}(\alpha ,\zeta ,a)=\overset{a}{\underset{i=0}{\sum }}\left(\frac{\left({\left(\alpha \cdot a\right)}^{\overset{n=\left(\zeta -1\right)}{\underset{i=0}{\sum }}n}\right)}{\overset{n=\left(\zeta -1\right)}{\underset{i=0}{\sum }}n!}\right)\cdot e^{\left(-\alpha \cdot a\right)},$$

where *ζ *= 6, *g* is a function of mosquito size, and *RH* is relative humidity. The mortality rate for each age interval can then be approximated as

(32) $$\beta_{N,m_{n}}=\left\{\begin{array}{cc} \frac{\log\left(\frac{\varpi_{N,m_{t_{2}}}}{\varpi_{N,m_{t_{1}}}}\right)}{\mathrm{\Delta t}} & \;\text{if}\;T<40\\ 3 & \;\text{otherwise} \end{array},\;\right\}.$$

If we assume that differences in adult mortality for *An. gambiae s.s.* and *An. arabiensis* can be explained by differences in body size, these BLLad curves can be used for both species. We explore this mortality model in [64].

#### AL adult mortality

A similar approach can be used for *An. arabiensis*. Using survival curves reported by Afrane et al. ([112], Figure two) (copyedited with g3data [113]), we can estimate mortality based on the daily maximum temperature. Because of the few data points, this approach is much more uncertain and should be considered experimental. The advantage of this mortality model is that the data are not estimated from a laboratory setting. The maximum temperature reflects some aspects, such as radiation, albedo, and humidity, of the environment in which mosquitoes live. In some of the results presented in part two, we use this model for adult survival.

(33) $$\begin{array}{ll} \alpha & =c_{1}-c_{2}\\ & \;\cdot \left(c_{9}+T_{\text{mod}}^{c_{3}}\cdot \left(T_{\text{mod}}^{c_{4}}-T_{\text{mod}}\cdot c_{5}-c_{8}\right)-c_{7}\cdot \frac{c_{10}^{\frac{T_{\text{max}}}{c_{11}}}}{c_{6}}\right)\\ & \;+e^{-\left(\frac{T_{\text{max}}}{5}\right)}\cdot c_{11} \end{array}$$

(34) $$T_{\text{mod}}=1+\frac{T_{\text{max}}+18}{11.10}.$$

Constants *c*_1,…,11_ are listed in Table 2. By setting *ζ *= 2 we can simplify the survival curve for *An. arabiensis* to

(35) $$\varpi_{N,m}\left(\alpha ,\zeta ,a\right)=\overset{a}{\underset{i=0}{\sum }}\left(1+\alpha \cdot a\right)\cdot e^{-\alpha \cdot a}.$$

**Figure 6:**
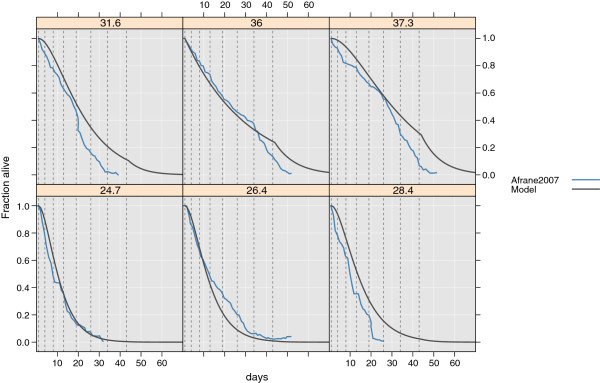
**Proportion of *An. arabiensis *surviving at daily maximum temperatures.** Estimated from Afrane et al. [112 ](blue line). Dashed vertical lines indicate the different age groups in the model (grey lines).

The corresponding curve is shown in Figure 6.

### Parametrization of air temperature

Paaijmans et al. discussed the importance of using indoor rather than outdoor temperature, to describe the environment for mosquitoes and parasites [114]. They included two studies that showed the relationship between indoor and outdoor temperature in Kenya [115] and Tanzania [116]. Here we add two additional studies, one from Kenya [48] and one describing the temperature in traditional and low-cost modern housing in the Eastern Cape, South Africa [117]. The data used to parametrize equation 36 came from; 1, Afrane et al. [48]; 2, Makaka and Meyer [117]; and 3, Paaijmans et al. [114-116] (*R*^2 ^= 0.89). It is clear that temperatures inside a house are more stable than outdoor temperatures. House type greatly influences daily temperature fluctuations [117,118], and the model used here might not be valid for all house types. While some studies have assumed that houses are always hotter than the surroundings [119], we approximate the indoor temperature as

(36) $$T_{\text{indoor}}=10.33+0.58\cdot T_{\text{air}}.$$

**Figure 7:**
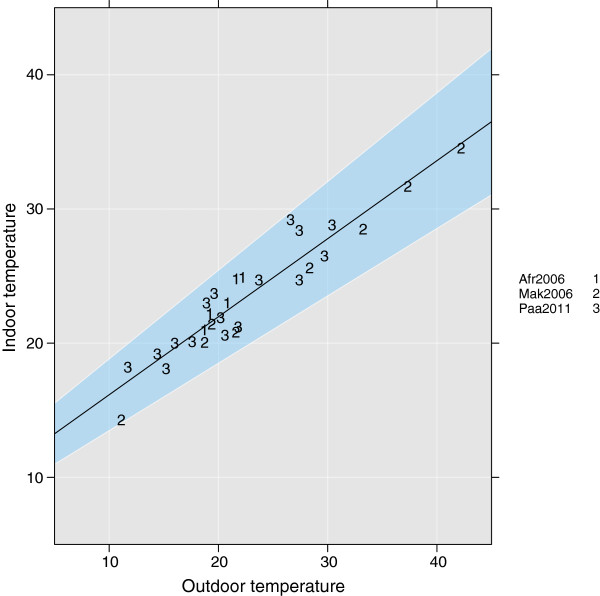
**Relationship between outdoor and indoor temperatures.** Numbers denote the study from which data were taken: 1, Afrane et al. [48]; 2, Makaka and Meyer [117]; and 3, Paaijmans et al. [114-116]. The blue area represents the 95% confidence interval, and the black line shows Eq. 36. *R*^2 ^= 0.89.

Since the data are based on maximum and minimum temperatures, the timing of the indoor temperature might be offset by a couple of hours. This is evident in a study by Makaka and Meyer [117], who delayed the maximum indoor temperature by a couple of hours compared to the environmental temperature. At present we do not account for this delay, since the diurnal temperature ranges will be correct even if we do not. The data and regression line are shown in Figure 7. Further studies on indoor compared to outdoor temperatures are needed to make this correction more accurate.

Hence, *T*_*air *_can be partly or fully replaced by *T*_*indoor *_, depending on the proportion of mosquitoes indoors.

It should be noted that we still do not include temperatures in resting places described by Holstein, such as holes in rocks and cracks in soil, covered pigsties, rabbit hutches, hen coops and dry wells [98], and by de Meillon ([120], under stones).

### Approximation of mosquito movement

The role of diffusion and advection in vector borne diseases have been explored in several papers [102,121-127]. Considering the gradual invasion of Brazil in the 1930s by *An. arabiensis*[60 ]it can be argued that movement of mosquitoes is important over decades. Here we include the active and passive transport of mosquitoes as fluxes across grid boundaries. Passive transport is movement of mosquitoes caused by wind, while active transport is movement due to flying. On shorter time scales the role of such movement will be limited. However, on long time scales it is necessary to allow mosquitoes to travel to allow them to establish in new locations.

Transport of mosquitoes is defined by fluxes (*s*^-1^) at the grid boundaries. In the model we allow fluxes from the eight neighboring grid points. A special case is implemented when a neighbouring cell is water. In this case, fluxes to water are reduced to 0.1*%* of the original flux to avoid large losses of mosquitoes along the coastline. Given strong winds from land to the ocean, such an assumption could lead to accumulation of mosquitoes along the coast. Conversely, allowing free movement to the ocean could lead to undesired loss of mosquitoes.

Since the movement of mosquitoes has a high computational cost, the spatial fluxes do not change the size calculations. This will introduce some minor errors when the movement of mosquitoes is low compared to their density, with larger errors if many mosquitoes are moved relative to their density. When a cell free of mosquitoes is colonized, the size is set to 3.05 mm.

The possible flight range of anophelines varies with food availability [128]. We do not include vegetation types in the model and hence it is hard to justify differences in flight performance based on, for example, land use. The dispersion coefficient describes how far mosquitoes can move in a day. We assume that the dispersion coefficient *D* is constant, independent of geographical location. For *An. gambiae s.s.* and *An. arabiensis*, real flight performance outside the laboratory of only a few hundred meters per day (approx. 300-700 m) has been reported [102,129,130]. In this experiment we subjectively chose *D*=30*mday*^-1 ^independent of age group. Anophelinae also travel with humans [131], which adds to the transport equation and makes the dispersion coefficient uncertain. Gillies noted that wind direction mostly has a minor effect on dispersal [129], while de Meillon [132] and Adams [133] reported distances of 2-4.5 miles (3-7 km) in the direction of the prevailing wind. Thus, it cannot be ruled out that wind plays a role on longer time scales. Hence, we express movement caused by wind as a function of 10-m zonal (*u*) and meridional (*v*) wind components (*ms*^-1^). This can be understood by considering the following example. For a constant *u*-wind of 10*ms*^-1^ and *v*-wind set to 0, mosquitoes will be moved a distance related to a scale factor *S*_*f*_, which is equal to the distance travelled at 20*ms*^-1^ to the east. For example, with *S*_*f *_= 750*mday*^-1^, the eastward distance traveled will be $S_{f}\cdot \frac{10{\text{m s}}^{-1}}{20{\text{m s}}^{-1}}375$ m in 1 day, but since each mosquito is not modelled individually, it would be more natural to describe this as a fraction moving a certain distance. Different wind directions and speeds will result in other distances/fractions and directions. *D* and *S*_*f *_are unknown tunable constants.

Since the species considered here are most active at night [22], movement will be suppressed between 06:00 and 18:00 h (local time) and amplified at night according to

(37) $$\kappa =\frac{{\left(\cos\left(\text{LT}\cdot \frac{\Pi }{12}\right)+1\right)}^{2}}{1.506925},$$

where *LT* is local time, $\overset{24}{\underset{0}{\int }}\kappa \approx 1$ and

$$\text{LT}\;=\;\left\{\;\;\begin{array}{cc} \text{UT}C_{\text{time}}\;+\;\frac{\text{longitude}}{15}\;-\;24 & \;\;\;\text{if}\;\text{UT}C_{\text{time}}\;+\;\frac{\text{longitude}}{15}\;>=\;24\\ \text{UT}C_{\text{time}}\;+\;\frac{\text{longitude}}{15}\;+\;24 & \;\;\;\text{if}\;\text{UT}C_{\text{time}}\;+\;\frac{\text{longitude}}{15}\;<=\;0\\ \text{UT}C_{\text{time}}+\frac{\text{longitude}}{15} & \;\;\;\text{otherwise}. \end{array}\right)$$

Transport of mosquitoes and mosquito sizes inside and outside a grid are defined by

(38) $$\frac{\delta m_{n}}{\mathrm{\delta t}}=\overset{1}{\underset{ı=-1}{\sum }}\overset{1}{\underset{ȷ=-1}{\sum }}\Phi_{ı,ȷ}m_{n,ı,ȷ}.$$

More specifically, during a time *Δt*, movement can be calculated as follows. On a day with no wind, transport is equal in all directions, *D *= 30*mday*^-1^, and the flux at a boundary is defined as

(39) $$\Phi_{ı,ȷ}=\kappa_{ı,ȷ}\mathrm{\Delta t}\cdot \frac{D}{\Delta d_{ı,ȷ}\cdot 24\cdot 60\cdot 60},ı=\{-1,1\},ȷ=\{-1,1\}$$

and transport *η*_*ı*,*ȷ*__*n *_is then equal to

(40) $${\eta_{ı,ȷ}}_{n}={m_{ı,ȷ}}_{n}\cdot \Phi_{ı,ȷ}.$$

In the presence of wind, we obtain additional transport as a function of zonal and meridional wind components.

### Mortality related to feeding

One factor that is often overlooked in malaria (mosquito) models is survival related to food availability (*P*(*B*)). Ye-Ebiyo et al. reported that maize pollen availability has a positive effect on larval (and hence mosquito) fitness [70,134]. Creating maps of plant types is beyond the scope of this study, and hence we chose not to account for mortality related to crops. However, we performed initial tests in which we included GlobCover Land Cover version V2.2 (European Space Agency [135]) to give a rough estimate of regions where increased fitness could be expected. The other source of food for female anophelines is blood. Compared to a starved mosquito, a mosquito that has had access to blood on days 1-3 has a theoretical flight distance that is increased by a factor of 6-7 [128]. Therefore, it is plausible that the higher (lower) the probability of finding a blood meal (*P*(*B*)), the higher (lower) is survival in the early life stages of adult mosquitoes. Bouma and Rowland reported higher parasite prevalence among children of families who kept cattle compared to those who did not [136], which can indicate either higher survival (older mosquitoes) or simply that some anophelines are attracted to cattle. If we assume that a newly emerged mosquito has a flight range of *Fr*_*m *_= 0.5*km*^2^*day*^-1^, the daily probability of finding a blood meal can be calculated as

(41) $$P(B)=\left\{\begin{array}{cc} \text{HBI}\cdot \rho_{\text{human}}\cdot Fr_{m}+(1-\text{HBI})\cdot \rho_{\text{bovine}}\cdot Fr_{m} & \;\text{if}\;\;P(B)<1\\ 1 & \;\text{otherwise} \end{array}\right\},$$

where *ρ*_*human *_and *ρ*_*bovine *_is the probability of finding a human and bovine source, respectively. *ρ*_*humans *_is defined as the human population density per *km*^2 ^multiplied by 0.1 (since a smaller area on a human is accessible) and *ρ*_*bovine *_is defined as the bovine density per *km*^2^, each with a user-defined threshold at which the density is so low that *P*(*B*) is virtually zero. Since *P*(*B*) is a conceptual parameter, it can be tuned.

Since blood meals, besides sugar meals, are important for the mobility [128] and survival of female anophelines [137], the success of a species is likely to be linked to the presence of the preferred host. The dominant blood source for *An. arabiensis* is bovine and human blood, while it is human blood for *An. gambiae s.s. *[138]. In reality there are strong indications that the human blood index is a dynamic quantity rather than a constant [139-142]. In the current implementation, *HBI* is a static number and hence there are probably errors related to this term. To find the probability of feeding on humans at each time step, we combine two data sets. Between 2000 and 2010 we use population densities from the Gridded Population of the World (GPW) [42], and for before 2000 and after 2010 we use growth rates from the Population Division of the Department of Economic, and Social Affairs of the United Nations Secretariat [143]. Since there are no projections of cattle densities, this quantity is time-invariant and based on Food and Agriculture Organization (FAO) 2005 estimates [44]. We are currently working to include time-varying cattle densities.

In the model, mortality caused by food limitations is a function of how many humans or cattle are available per mosquito and the human blood index. We assume that *HBI* is time- and space-invariant, and only depends on the species. For simplicity we chose available humans to be humans who are not sleeping under a bed net. In the simulations presented here, we set bed net usage to zero, and hence the results represent mosquito distribution without interventions. Bayoh et al. hypothesized that the survival of the different species is related to the availability of the preferred host [9]. The daily mortality rate caused by limited human blood is expressed as

(42) $$\beta_{h,m}=\text{max}\left(1-\frac{\frac{30}{\text{HBI}}\sum h}{\overset{n=\infty }{\underset{n=2}{\sum }}m_{n}},0\right).$$

The functional form of of equation 42 can be seen in Additional file 3.

**Figure 8:**
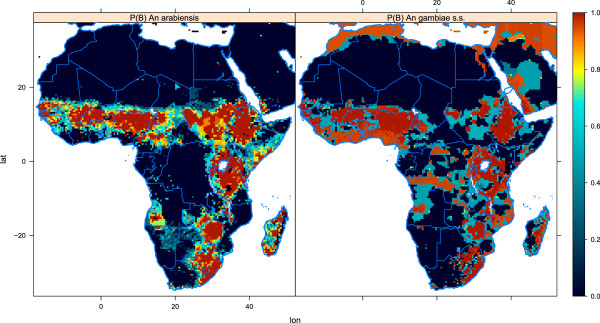
**Probability of finding a blood meal for *An. arabiensis *(*HBI = 0. 4 *) and *An. gambiae s.s. *(*HBI = 0. 95*) with zero bed net coverage.**

Figure 8 shows the probability of finding a blood meal for the sibling species on January 1, 1999.

## Results and discussion

### Sensitivity experiments

Sensitivity experiments are useful in understanding which parameters are important for the success of *An. arabiensis* and *An. gambiae s.s.* and which are important for malaria transmission. Classical sensitivity analysis investigates the robustness of a study when parameters are estimated from statistical modelling. Our model uses parametrization schemes to represent the influence of the environment on the two species. We show how the model responds to changing temperature, humidity, mosquito size, dispersion and the probability of finding blood. This approach does not allow us to directly measure the robustness of each parametrization scheme, but gives us an insight into which external factors influence the model and where it is of importance to have improved parametrization schemes. We use the term sensitivity experiments for this analysis.

#### Settings

To demonstrate some of the capabilities of the model, we set up a series of experiments. Some aspects are best visualized as a one-dimensional model (time and age), while other features are shown using a spatial domain (time, age, and space). For the one-dimensional experiments, the water temperature is set to the air temperature, except for temperature greater than 33°C, for which we set temperature to 33°C. This modification is required since pupae and fourth instar larvae will not develop below 18°C or above 34°C [144]. The results are therefore less robust when temperature is greater than 33°C. Unless otherwise stated, we use size-dependent mortality, correction for indoor temperature, the KBLL method to estimate mortality in the aquatic stages, correction for the development rate in the aquatic stages depending on the ratio of each species, and movement of mosquitoes (in the spatial cases).

Sensitivity to temperature, relative humidity and mosquito size (TempHumSize)

The age-dependent mortality is influenced by temperature, relative humidity and mosquito size [Eq. (32)]. This experiment explores how the dynamics of malaria is sensitive to temperature, relative humidity and mosquito size (measured as mm). We assume that no births occur to isolate the effect of the transmission process, and consequently constant mosquito body size in the course of integration, but include mortality and the biting rate. In this experiment we assume that only one species is present (since the main competition occurs in the aquatic stages). This sensitivity test is designed to observe how the proportion of mosquitoes becomes infected as a function of temperature, relative humidity and mosquito size, given that we start with 1000 newly emerged mosquitoes, with *m*_1 _= 1000 and *m*_2-9 _= 0 as the initial conditions. In this experiment, 1% of the human population is infectious for *Plasmodium falciparum*. Mosquitoes are infected with an efficiency of 100%, meaning that biting an infectious human results in gametocyte transmission to the mosquito. In practice, this would be the same as saying that 10% of humans were infectious and gametocyte transmission had an efficiency of 10%. We also neglect the effect of heterogeneous biting. This is the only experiment in which we model the proportion of infectious mosquitoes explicitly. The modified equations describing the transmission process are described in [64].

The rate of sporozoite development within mosquitoes is expressed as [5]

(43) $$\text{pf}={\left(a+\frac{b}{e^{{\left(T_{\text{air}}\right)}^{c}-d}}\right)}^{-1},$$

where a = 9.5907, *b *= 0.0051029, *c *= 0.7349, and *d *= 17.0325. This expression was derived from the figure in MacDonald page 119 [5] using g3data [113], and fitted using non-linear least-squares [145].

The gonotrophic cycle and biting rate are defined in Eq. (26).

**Figure 9:**
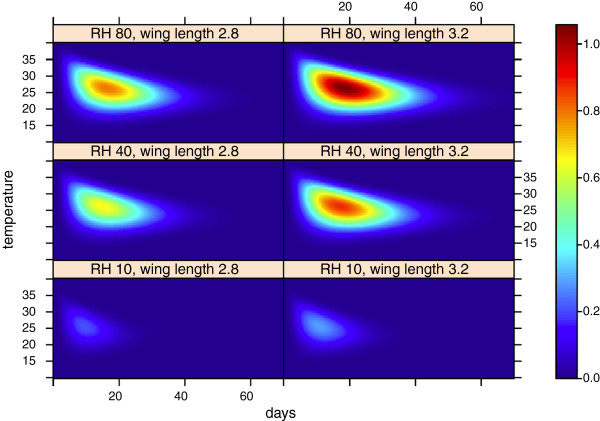
**Percentage of 1000 mosquitoes that are infectious after *x *days. **The *y*-axis represents temperature in degrees centigrade. The model is integrated at two mosquito sizes (2.8 and 3.2 mm for wing length) and three relative humidity values.

The integrations are repeated with different combinations of temperature and relative humidity. This is a simple representation of gametocyte transmission to mosquitoes and is an idealized approach for exploring the proportion of mosquitoes (of the original 1000) that would become infected under different temperature, RH and mosquito size. Figure 9 shows how the percentage of infectious mosquitoes changes with temperature, RH and mosquito size. Lyimo and Koella reported that the largest mosquitoes were less likely to have sporozoites, but had more oocysts than smaller mosquitoes [54]. They attributed this to increased mortality in the presence of many oocysts, an effect that is not included in our model. Figure 9 shows that the potential percentage of infected mosquitoes is sensitive to all three parameters in the model. Although higher survival has been attributed to body size in dry [7,51] and semi-arid environments [55], the advantage or disadvantage of a larger body has been poorly described in saturated environments. Therefore, the sensitivity to body size at 80% RH should be interpreted with care. According to the model, temperature is not the only factor that governs the transmission of malaria (in areas with no interventions); humidity and how mosquitoes adapt to dehydration stress are also important factors. The most efficient transmission, expressed as the integral, with respect to days, occurs at 25°C at 40% and 80% RH, and at 24.5°C at 10% RH, independent of mosquito size.

These results should be viewed in light of recent findings by Paaijmans et al. that optimal transmission occurs at lower temperatures [4].

Sensitivity to temperature and carrying capacity (TempCar)

The aim of this sensitivity test was to investigate how carrying capacity and temperature determine the relative proportion of *An. arabiensis* and *An. gambiae s.s.* We set the relative humidity to 80% and the probability of getting a blood meal to one. We assumed that the soil was saturated and we varied the temperature between 16 and 38°C (with corrections over 33°C for water temperature) and the carrying capacity between 0.0625 and 125 *mgkm*^-2^.

Carrying capacity in the aquatic stages influences larval growth and adult survival. While *An. arabiensis* invests more time in growth than *An. gambiae s.s.*, the former develops a larger body, and consequently has the potential to oviposit more eggs than the latter. If the two species experience the same mortality rate in the aquatic stages, more *An. gambiae s.s.* will emerge, but over time *An. arabiensis* can face this challenge by outnumbering the eggs of *An. gambiae s.s.* in the habitat. Thus, we are interested in testing how the carrying capacity in the aquatic stages alters the relative proportion of each of the adult species. In this model we only consider the competition between these two species, and hence neglect other competing species [146].

**Figure 10:**
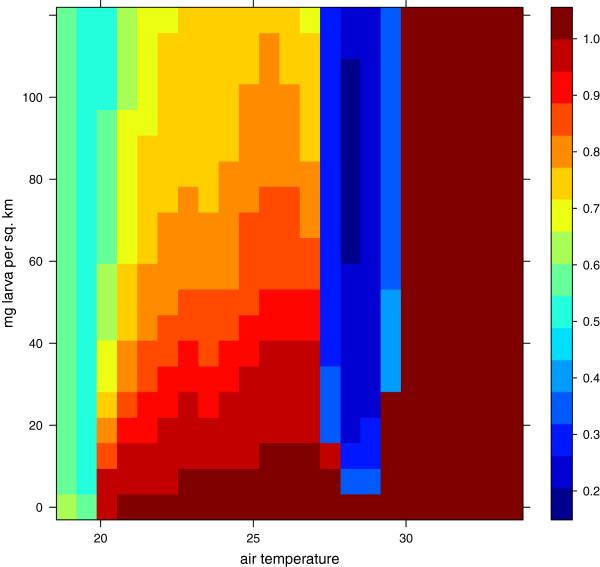
**Fraction of *An. arabiensis * as a function of air temperature and carrying capacity.** The water temperature is set to the same value as the air temperature unless the temperature is greater than 33°C (at which most pupae would not develop into adults [144]). In this case the water temperature is set to 33°C, but the productivity will remain low. The fraction of *An. gambiae s.s.* is one minus the fraction for *An. arabiensis*.

As observed in Figure 10, *An. gambiae s.s.* dominates between 27 and 30°C. This is the effect of the development rate modifications described by Kirby et al. [72] and Paaijmans et al. [8] (Figure 2 and “Species-dependent mortality (KBLL)”). Interestingly, the dominance of *An. arabiensis* is most pronounced in the drier simulations, meaning that high competition, compared to adult survival, is favourable for this species. This can be attributed to the strategy of larger body size and higher egg production. Lehmann et al. found that *An. arabiensis* dominated during the dry season, while *An. gambiae s.s.* dominated in the rainy season [57]. The advantage of *An. arabiensis* in crowded breeding places might be one factor contributing to the shift in species composition as the surface area of puddles starts to shrink.

Sensitivity to temperature and the probability of finding blood (pBlood1D)

This experiment shows how the model responds to changes in the probability of finding a blood meal, which influences the rate at which mosquitoes can oviposit and increases energy consumption if hosts are hard to locate. If, for example, cattle are easier to find compared to humans, *An. arabiensis* will potentially use less energy per batch of eggs and will also be able to utilize breeding sites at a higher rate than *An. gambiae s.s.* It is also possible that *An. arabiensis* uses cattle for navigation [147]. Over time, such differences might lead to dominance by one species. In this experiment, we varied the probability of finding blood, *P*(*B*), for *An. arabiensis* from zero to one, as well as varying the temperature as described for TempCar.

We set the probability of finding blood to one for *An. gambiae s.s.*, independent of the probability of *An. arabiensis* finding a blood meal. This is a purely theoretical experiment designed to demonstrate a concept. The probability of finding blood is varied between zero and one for *An. arabiensis*. The scenario in which *P*(*B*) = 1 for *An. gambiae s.s.* and zero for *An. arabiensis* is not a realistic scenario, but the difference in *P*(*B*) is grounded in differences in their feeding behaviour, whereby *An. arabiensis* can utilize cattle more efficiently than *An. gambiae s.s.*, for example.

**Figure 11:**
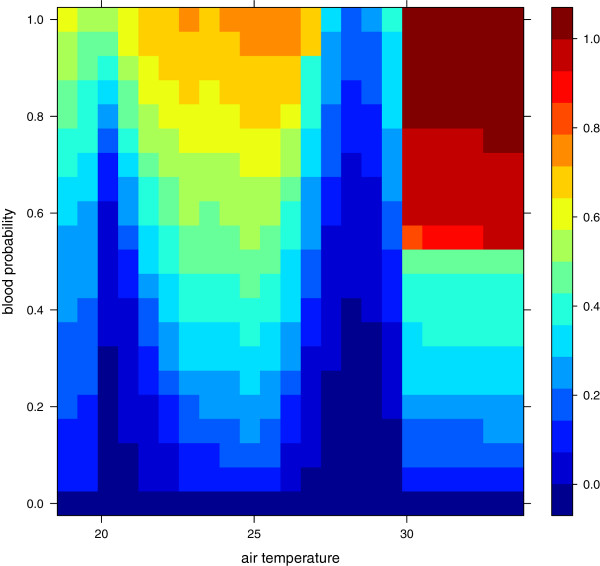
**Fraction of *An. arabiensis *as a function of air temperature and probability of finding a blood meal.** The fraction of *An. gambiae s.s.* is one minus the fraction for *An. arabiensis*.

Figure 11 shows the relative fraction of *An. arabiensis*. In addition to the pattern observed in Figure 10, it is also evident that if *P*(*B*) is low for *An. arabiensis*, *An. gambiae s.s.* dominates. *P*(*B*) can be interpreted as a parameter that describes both the probability of finding blood for reproduction and survival, and the energy spent in the search for a blood meal. For example, easy access to cattle might give *An. arabiensis* an advantage in exploiting breeding sites, which could lead to suppression of the number of *An. gambiae s.s.* if increased use of bed nets reduces the effective human population or causes higher mortality of anthropophilic species. This mechanism might help to explain the decline in *An. gambiae s.s.* observed by Bayoh et al. [9].

#### Sensitivity to the probability of finding blood in a spatial domain (pBlood2D)

This experiment is similar to pBlood1D, but this time we integrate the model for 5 years over the African domain. The experiment consists of two runs, for which the first has *P*(*B*) similar to Figure 8 and the second has *P*(*B*)=1 over all land areas for both species. The population density is space-invariant at 400 *humans*/*km*^2 ^(remember that the number of mosquitoes is limited by the number of hosts). Thus, the only limitation in this experiment is the physical environment (air and water temperatures, relative humidity, wind and run-off), which is updated every 3 h. The initial conditions for the mosquito populations were the same for the two runs.

Even though we have stated that the probability of finding blood *P*(*B*) is an expression of the cost of finding a host, it might well be that *P*(*B*) also includes a component that describes the environment shaped by cattle and humans. Therefore, it should be noted that it is difficult to distinguish between the true probability of finding blood and the environmental changes caused by the presence of humans or cattle.

**Figure 12:**
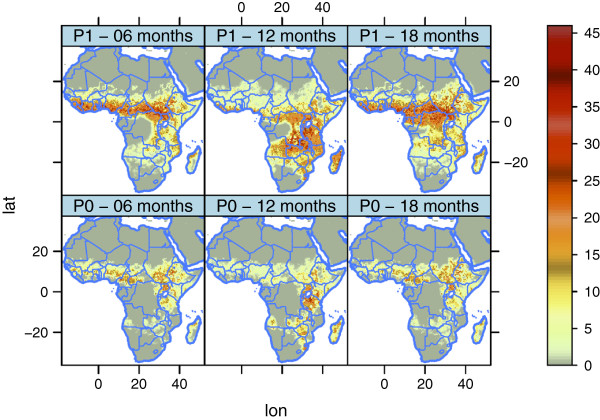
**Square root of number of*An. arabiensis* per*km***^***2 ***^**in the two pBlood2D experiments.** In P0 we used realistic values of the probability of finding blood, *P*(*B*), while in P1 the probability of finding blood was set to 1, independent of the location. See the text for further details.

**Figure 13:**
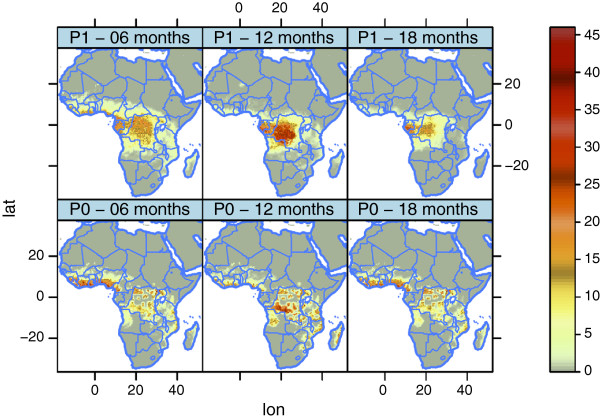
**Square root of the number of *An. gambiae s.s. * per*km***^***2 ***^**in the two pBlood2D experiments.** In P0 we used realistic values of the probability of finding blood, *P*(*B*), while in P1 the probability of finding blood was set to 1, independent of the location. See the text for further details.

Under the scenario of equal probability of finding blood for the two species, *An. gambiae s.s.* loses the competition after 5 years (Figures 12 and 13), probably because of the greater reproductive potential of *An. arabiensis*. The only strongholds left for this species are DRC, Congo, and Gabon. Hence, the strategy of *An. arabiensis* to develop a larger body, produce more eggs, and possibly reduce adult mortality at the cost of spending more time in the aquatic stages is successful when access to blood is unlimited. *An. arabiensis* has extended its distribution as far north as the southern tip of Western Sahara. While the original set-up of the model (P0) limits the distribution of *An. gambiae s.l.* to approximately 17°N in the Sahel, the experiment with *P*(*B*)=1 (P1) has a distribution up to 22°N in Mali, Niger, Chad and Sudan. This is in line with observations of the northerly limit of *An. gambiae s.l. *[148-150]. The lack of *An. gambiae s.l.* north of 17° in the original set-up (P0) might be a result of the way the model is formulated. The population density is calculated within a box of approximately 50 *km*×50 *km*. It might well be the case that pockets of higher population/cattle densities within this box could sustain a mosquito population. This is not resolved in the model. It is also worth mentioning the study by De Meillon [151] of the anophelines of Namibia, which revealed that *An. gambiae s.l.* is present in large parts of the country. The original set-up (P0) allows sustainable mosquito populations in Namibia, while the density of *An. gambiae s.l.* in P1 is more comparable to the observations of De Meillon. The problems of capturing the distribution of *An. gambiae s.l.* in Namibia may originate from the problems of resolving pockets of high host density or changes in cattle density and distribution at the time of the study compared to the present day [43,44,152].

It is also worth mentioning that the density of *An. gambiae s.l.* in South Africa is not very sensitive to the probability of finding a blood meal. Hence, the distribution of *An. gambiae s.l.* is mainly restricted by climate according to the model.

Figures 12 and 13 show the distribution and density of *An. arabiensis* and *An. gambiae s.s.* under realistic (P0) and space-invariant (P1) *P*(*B*) after 6, 12, and 18 months. The integration was started on January 1 and the model was run for 5 years.

#### Mosquito transport (mosqTran)

The purpose of this experiment was to demonstrate how the initial conditions and competition influence the distribution of *An. gambiae s.s.* and *An. arabiensis*. To explore the theoretical dispersion distance and the influence of the initial conditions, we set up a simple experiment. In mosqTran(a) the model was initialized with *An. arabiensis* at -4.494381°E, 14.0154°N (Sahel), and *An. gambiae s.s.* at -4.494381°E, 6.502846°N (Cte d’Ivore, Ivory Coast) on January 1, 1989. The second experiment, mosqTran(b), had the same setup, but without *An. arabiensis*.

The purpose of this demonstration was to show the importance of mosquito movement and how new areas can or cannot be colonized. In a model in which movement is restricted, the vector range would also be restricted by the initial model conditions. For example, if only one point was specified for mosquitoes at the beginning of the integration, only the same point would have mosquitoes after 100 years. With dynamic movement the mosquitoes could colonize new areas if the environmental conditions, or the probability of finding blood, change over time.

**Figure 14:**
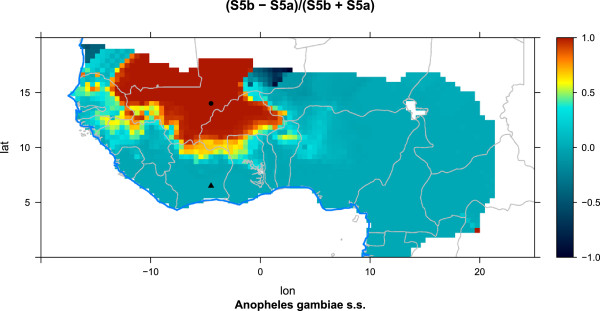
**Relative change in dispersal (mean over 5 years) for *An. gambiae s.s. *with (mosqTran(a)) and without (mosqTran(b)) *An. arabiensis*.** The black solid circle and triangle indicate the initial position of *An. arabiensis* and *An. gambiae s.s.*, respectively.

Figure 14 shows the relative difference in *An. gambiae s.s.* distribution in the two experiments. It is evident that in the presence of *An. arabiensis*, *An. gambiae s.s.* fails to colonize large parts of Mali and Burkina Faso. It can be argued that this is not a result of the initial conditions, but of competition. Additional file 4 illustrates why this is indeed a result of the initial conditions, although the initial conditions would not play a role in the absence of competition.

**Figure 15:**
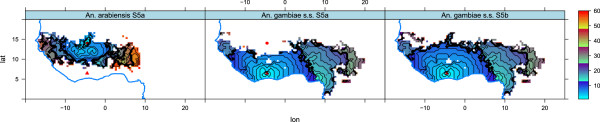
**Number of months required to reach a density of *20 mosquitoes/km***^***2***^**.** Panel 1 (left to right) represents *An. arabiensis* in experiment mosqTran(a), panel 2, *An. gambiae s.s.* with the presence of *An. arabiensis* (mosqTran(a)), and panel 3, *An. gambiae s.s.* with no competition (mosqTran(b)). The red solid circle and triangle indicate the initial position of *An. arabiensis* and *An. gambiae s.s.*, respectively.

Figure 15 shows the number of months required to reach a density of 20 *mosquitoes*/*km*^2^. It is interesting to note that dispersal occurs in pulses. The dispersal of *An. arabiensis* is slower than that of *An. gambiae s.s.*, probably because of the drier conditions in the Sahel and *An. gambiae s.s.* reached the area before *An. arabiensis* (Figure 15). The simulations show that establishment in an already occupied area is a much slower process compared to the case of no competition. From the simulations we can also speculate on whether the dominance of one species can act as a barrier to genetic flow, like a mountain range or dessert. This also raises some questions regarding whether hibernation or dispersal is the mechanism behind the dominance of the *An. gambiae s.s.* M form in parts of Mali. Although there are strong indications that the *An. gambiae s.s.* M undergoes aestivation during the dry season [57,58,71], it is also possible that the persistence of the *An. gambiae s.s.* M form in the Niono district in Mali can serve as a refuge during the dry season [153]. In both cases the M form receives a kick-start at the beginning of the rainy season, and might slow down the dispersal of *An. arabiensis* and the S form of *An. gambiae s.s.* A similar mechanism could contribute to the dominance of *An. arabiensis* in Ethiopia in the Turkana district, where the presence of *An. arabiensis* prevents rapid invasion by *An. gambiae s.s.*

## Conclusions

We developed a model to predict the presence and abundance of *An. arabiensis* and *An. gambiae s.s.* The model is age-structured and includes mosquito dispersal.

Sensitivity tests showed that as well as temperature, relative humidity and mosquito size are important factors in malaria transmission. The result for body size is in line with several studies [7,51,54,55,88,154] and thus the model captures some of the aspects related to higher survival among larger individuals. Note that we have not accounted for the higher metabolism in large mosquitoes [71], which might reduce survival under warm and dry conditions. There are also contrasting results with respect to body size and egg production [155]. It is likely that there is an optimum size that depends on the environment and is a function of temperature and humidity. Currently there are few results to back up this statement. However, Sanford et al. found significant differences in *Anopheles gambiae s.s.* wing length between Mali and Guinea-Bissau [156].

We show that relative humidity can be important for malaria transmission. Several models have neglected the role of (relative) humidity [29,157] and it is true that desiccation might not be a driver of mortality at moderate humidity (>70%?). The main argument for leaving out this parameter is the corresponding reduction in model complexity. As long as rainfall drive the carrying capacity, mosquito numbers will be restricted at lower humidity (no rain), and as a consequence the resulting number of mosquitoes can be limited for the wrong reasons, but with the correct result. For example, Ermert et al. [28] handle this deficiency by reducing vector survival during dry atmospheric conditions, defined as a function of 10-day accumulated rainfall. More studies on the survival of *An. gambiae s.l.* in relation to size and relative humidity in the range 5-40% are needed for more confidence in the role of humidity in the survival of *An. gambiae s.l.*

Assumption of exponential mortality has several advantages (see Figure 5 for examples of models in which exponential mortality is used). The model becomes fast to solve and it is easier to analyse the equations analytically. However, several studies have shown that mortality of *An. gambiae s.l.* is not exponential, and that inclusion of an age dimension alters the expected outcome of interventions targeted to reduce the vector population [50]. Therefore, we believe that models in which age-dependent mortality is assumed should be further explored. The sensitivity tests also suggest that carrying capacity within a restricted area plays a role in the distribution of *An. arabiensis* and *An. gambiae s.s.* The true carrying capacity is hard to estimate on a continental scale and thus relies on qualified guesswork taking into account rainfall, groundwater and soil saturation, for example. Carrying capacity influences not only the relative distribution of the two species but also the total number of mosquitoes. To correctly estimate the biting rate, a correct estimate of carrying capacity is required, and thus more work is needed to parametrize puddle formation. It should also be noted that no current large-scale models can describe the formation of puddles as rivers retreat, as described by Animut et al. [158].

Experiment pBlood2D showed how the model responds to the parameter *P*(*B*), the probability of finding a blood meal. *P*(*B*) is important in describing a realistic distribution of *An. arabiensis* and *An. gambiae s.s.* Thus, we hypothesize that the large-scale distribution of bovines is key to the success of *An. arabiensis*. Likewise, large-scale human density favours the presence of *An. gambiae s.s.*

Finally, experiment mosqTran showed how the initial conditions influence the dispersal of *An. gambiae s.s.* (and *An. arabiensis*). The distribution of *An. gambiae s.s.* changes dramatically with the presence of *An. arabiensis*, and thus the initial model conditions are highly relevant for correct description of the distribution of the two species. When rainfall is highly seasonal, the first come, first served principle seems to be important for the success of a species in drier conditions. Whether or not this plays a role in the evolution of aestivation in *An. gambiae s.s.* M form [57] is a question that should be further investigated.

The strong influence of initial conditions on dispersal of the *An. gambiae* complex is not irrelevant when assessing the impact of climate change, since vectorial capacity varies between species.

The availability of mosquito models allows researchers to build on and improve our understanding of the role of the *An. gambiae* complex in malaria transmission. We hope to refine the model as new data on mosquito biology become available, and to incorporate the effects of interventions.

## Competing interests

The authors declare that they have no competing interests.

## Authors’ contributions

The work presented here was carried out as a collaboration between all the authors. BL and AS defined the research theme. TML designed methods and mosquito experiments, performed the model runs, analysed the data, interpreted the results and wrote the paper. DK, AS and TML designed and evaluated the regional climate simulations. EL provided input with respect to the malaria situation in Ethiopia, which in turn was used in the model formulation. All the authors have contributed to, seen and approved the manuscript.

## Supplementary Material

Additional file 1 Density of mosquitoes under different predation regimes and temperatures. Click here for file

Additional file 2 Idealized puddle model Click here for file

Additional file 3 The functional form of of equation 42. Click here for file

Additional file 4 **A note on how initial conditions can influence the spatial distribution of*An. gambiae s.l.*** Click here for file
