# Supplementary figures and images for: A dynamic model of some malaria-transmitting anopheline mosquitoes of the Afrotropical region. I. Model description and sensitivity analysis
Source: Malar J. 2013 Jan 23;12:28. doi: 10.1186/1475-2875-12-28 (PMC3664083; doi:10.1186/1475-2875-12-28)

$$100 * (cPred(0) - cPred(1))/cPred(0)$$

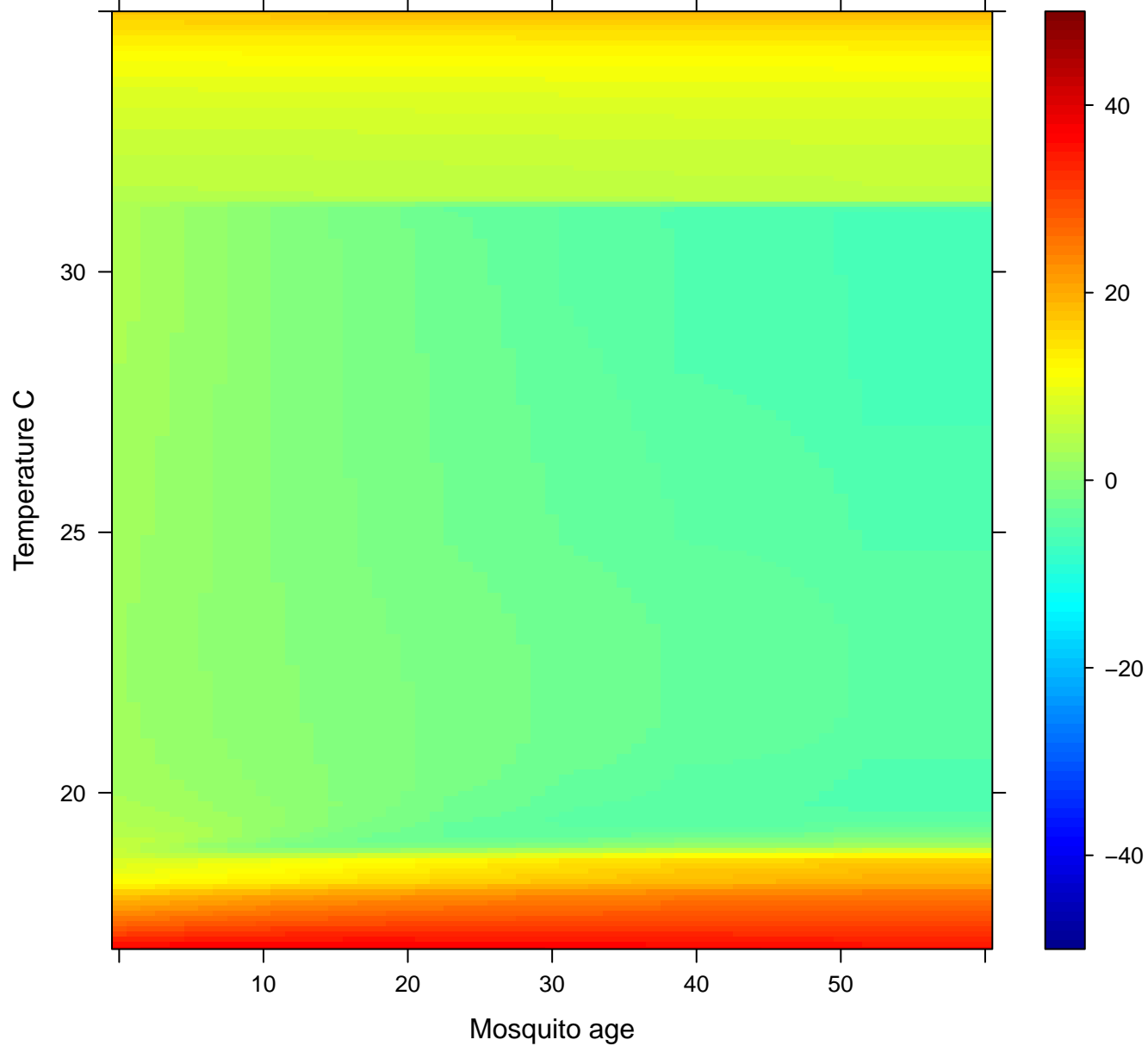

Supplement: Additional file 1 — Density of mosquitoes under different predation regimes and temperatures. [file 1475-2875-12-28-S1.pdf]

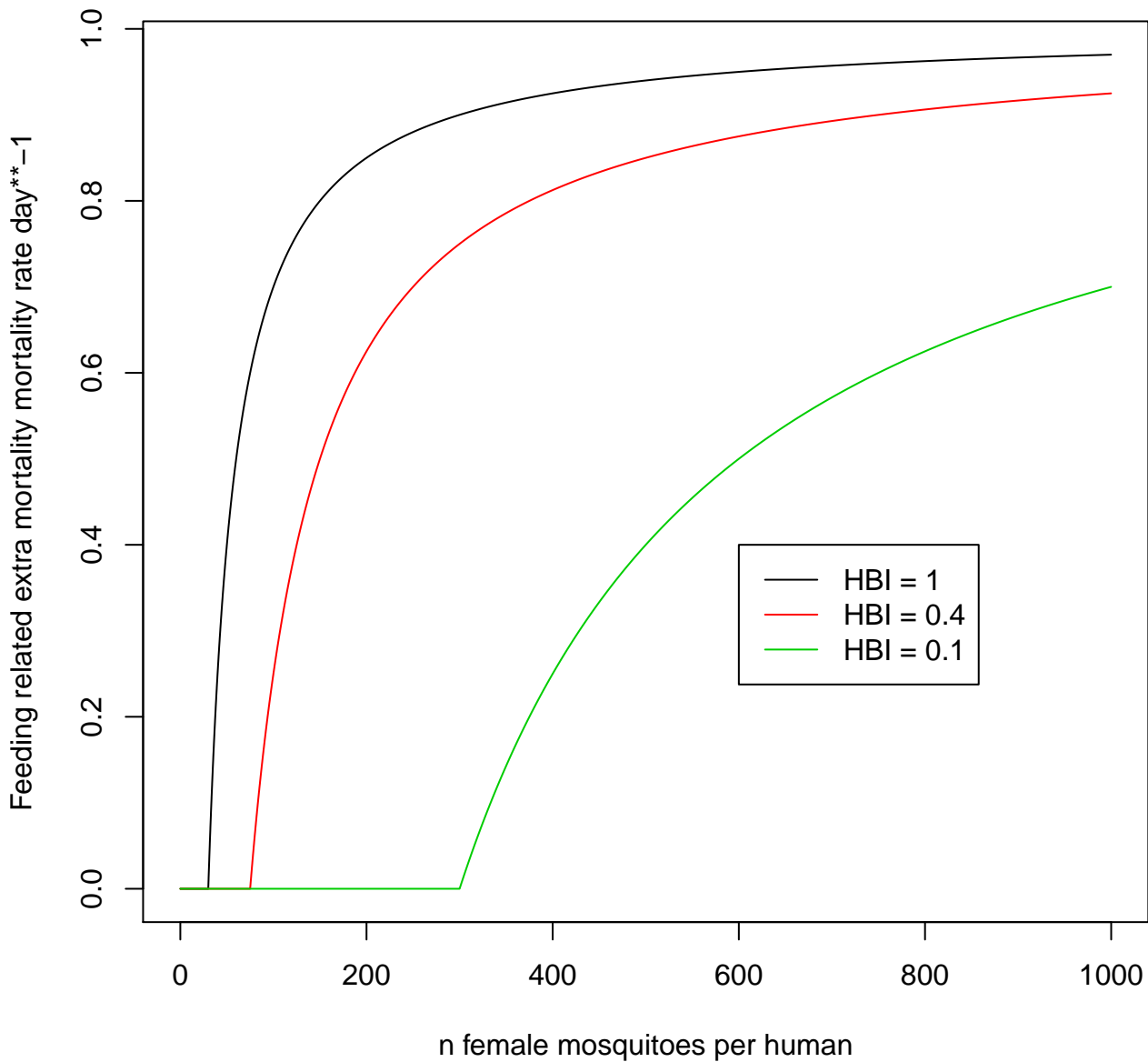

Supplement: Additional file 3 — The functional form of of equation 42. [file 1475-2875-12-28-S3.pdf]
