# Supplementary material for: A dynamic model of some malaria-transmitting anopheline mosquitoes of the Afrotropical region. I. Model description and sensitivity analysis
Source: Malar J. 2013 Jan 23;12:28. doi: 10.1186/1475-2875-12-28 (PMC3664083; doi:10.1186/1475-2875-12-28)
Supplement: Additional file 2 — Idealized puddle model [file 1475-2875-12-28-S2.pdf]

$$V_p(t) = \begin{cases} V_p(t-1) + Ro(t) - I(t) - E(t) + Rf(t) & \text{if } h < h_{max} \\ V_p(t) & \text{otherwise} \end{cases}$$

Where
t = time
V_p = volume of the puddle (m^3)
Ro = Run-off to+ the puddle (m^3)
I = infiltration (m^3)
E = evaporation (m^3)
Rf = rainfall (m^3)
h = depth of puddle (m)
h_{max} = maximum depth of puddle (m)
