# Supplementary material for: A dynamic model of some malaria-transmitting anopheline mosquitoes of the Afrotropical region. I. Model description and sensitivity analysis
Source: Malar J. 2013 Jan 23;12:28. doi: 10.1186/1475-2875-12-28 (PMC3664083; doi:10.1186/1475-2875-12-28)
Supplement: Additional file 4 — A note on how initial conditions can influence the spatial distribution ofAn. gambiae s.l. [file 1475-2875-12-28-S4.pdf]

1

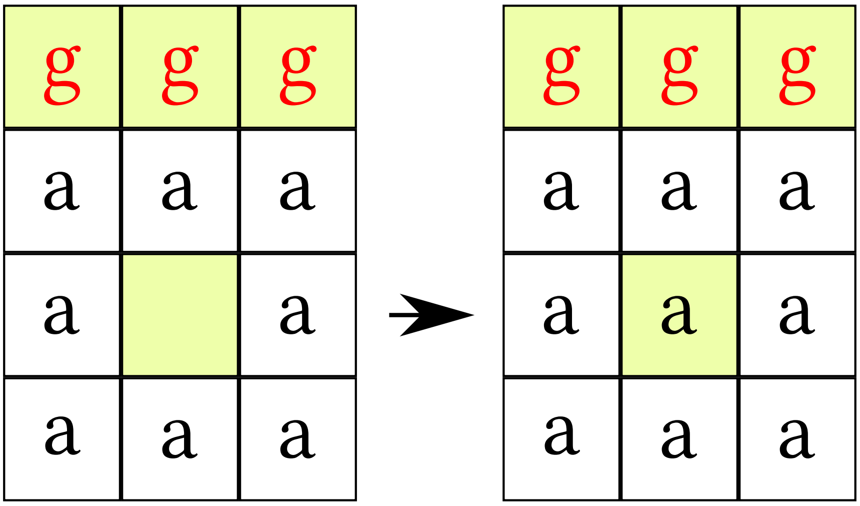

2

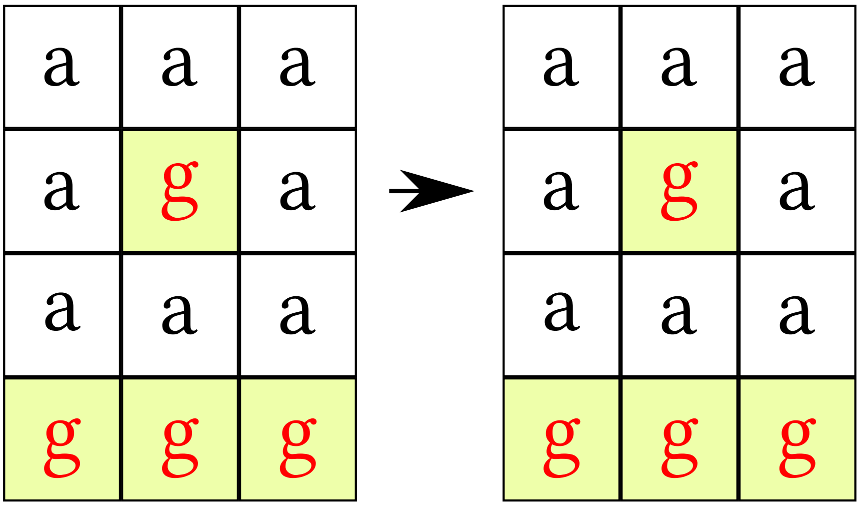

Why initial conditions and not competition?

The green areas indicate grid cells which are more suitable for An. gambiae s.s., red g indicate An. gambiea, while black a, indicate An. arabiensis.

In example 1 we initialise the model as shown to the left. After some time, An. arabiensis has occupied the cell which is most suitable for An. gambiae s.s; An gambiae s.s. is not able to pass the "wall" of An. arabiensis, and thus can not occupy this area.

In example 2, we initialise the model with An. gambiae s.s. in the suitable center area. In this case, An. arabiensis is not able to outcompete An. gambiae s.s. in this location.

Depending on the initial conditions, this cell can either have An. gambiae s.s., or An. arabiensis (or both). Competition does indeed play a role, but in this case the initial conditions are more important.
